# Self‐prioritization is supported by interactions between large‐scale brain networks

**DOI:** 10.1111/ejn.15612

**Published:** 2022-02-03

**Authors:** Alla Yankouskaya, Jie Sui

**Affiliations:** ^1^ Department of Psychology Bournemouth University Poole UK; ^2^ School of Psychology University of Aberdeen Aberdeen UK

**Keywords:** default mode network, emotion prioritization, frontoparietal network, large‐scale networks, salience network, self‐prioritization

## Abstract

Resting‐state functional magnetic resonance imaging (fMRI) has provided solid evidence that the default mode network (DMN) is implicated in self‐referential processing. The functional connectivity of the DMN has also been observed in tasks where self‐referential processing leads to self‐prioritization (SPE) in perception and decision‐making. However, we are less certain about whether (i) SPE solely depends on the interplay within parts of the DMN or is driven by multiple brain networks and (ii) whether SPE is associated with a unique component of interconnected networks or can be explained by related effects such as emotion prioritization. We addressed these questions by identifying and comparing topological clusters of networks involved in self‐and emotion prioritization effects generated in an associative‐matching task. Using network‐based statistics, we found that SPE controlled by emotion is supported by a unique component of interacting networks, including the medial prefrontal part of the DMN (MPFC), frontoparietal network (FPN) and insular salience network (SN). This component emerged as a result of a focal effect confined to few connections, indicating that interaction between DMN, FPC and SN is critical to cognitive operations for the SPE. This result was validated on a separate data set. In contrast, prioritization of happy emotion was associated with a component formed by interactions between the rostral prefrontal part of SN, posterior parietal part of FPN and the MPFC, whereas sad emotion reveals a cluster of the DMN, dorsal attention network (DAN) and visual medial network (VMN). We discussed theoretical and methodological aspects of these findings within the more general domain of social cognition.

## Graphical abstract

Self‐related processing is a product of information flow between default mode network, frontoparietal network and salience networks. The MPFC node of default mode network may play the central hub role in the activation of self‐representation through orchestrating interactions between these networks. The self‐prioritization effect could not simply be explained by general effects such as prioritizations of positive or negative emotion information.

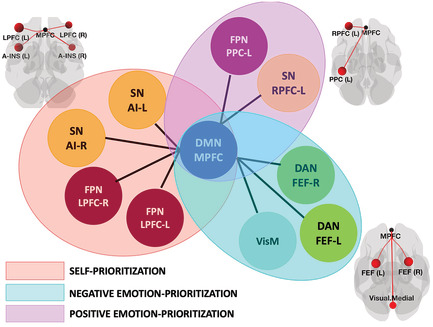

**Abbreviations**
ACC: anterior cingulate cortex
aCompCor: anatomical component‐based noise correction method
AI: anterior insula
ART: artefact detection toolbox
BOLD: blood oxygenation level dependent
CCN: cognitive control network
CEN: central executive network
CONN: functional connectivity toolbox
CSF: cerebrospinal fluid
DAN: dorsal attention network
DLPFC: dorsolateral prefrontal cortex
DMN: default mode network
dmPFC: dorsomedial prefrontal Cortex
FC: functional connectivity
FEF: frontal eye field
fMRI: functional magnetic resonance imaging
FPN: frontoparietal network
FWE: familywise error
GLM: general linear model
GSR: global signal regression
HCP: Human Connectome Project
ICA: independent component analysis
IPL: inferior parietal lobule
lFPN: left frontoparietal network
LP: lateral parietal
LPFC: lateral prefrontal cortex
LpSTS: left posterior superior temporal sulcus
MNI: Montreal Neurological Institute
MPFC: medial prefrontal cortex
NBS: network‐based statistics
PCC: posterior cingulate cortex
PPC: posterior parietal cortex
QC‐FC: quality‐control functional connectivity
rFPN: right frontoparietal network
ROI: region‐of‐interest
RPFC: rostral prefrontal cortex
RRC: region‐of‐interest to region‐of‐interest connectivity
SN: salience network
SNR: signal‐to‐noise ratio
SPE: self‐prioritization effect
SPM12: Statistical Parametric Mapping, Version 12
VMN: visual medial network
vmPFC: ventromedial prefrontal cortex

## 1 INTRODUCTION

The question of how the brain computes information related to ourselves has been of research interest for over three decades (see for review Frewen et al., 2020; Northoff, 2016). Despite substantial recent progress in disentangling neural substrates involved in self‐prioritization effects (SPE) (for review, see Sui & Humphreys, 2017), our understanding of the connectivity of these substrates underlying this complex brain function remains uncertain. This study makes the step forward in discovering neural properties of information processing biases for self compared with other social entities using a large‐scale brain network approach.

The way self‐relevant stimuli guide us through everyday perception is consistently described in the literature as effects gaining quicker access to visual awareness (Macrae et al., 2017), habit (Verplanken & Sui, 2019), engaging attention (Sui & Rotshtein, 2019), driving behaviours (Desebrock et al., 2018) and facilitating performance (Golubickis et al., 2017). These effects are conceptualized in ‘self‐prioritization’ as an umbrella term indicating biased information processing flow for items associated with self compared with items related to familiar or unfamiliar others. The SPE is robust and well replicated in multiple independent research using various experimental paradigms (Cunningham & Turk, 2017; Klein, 2012; Lee et al., 2021; Sun et al., 2016).

A large body of task‐based functional magnetic resonance imaging (fMRI) research has been devoted to studying the neural basis of the SPE, mainly focusing on brain regions. For example, it was suggested that the causal coupling between the medial prefrontal cortex (MPFC) and the left posterior superior temporal sulcus (LpSTS) facilitates information flow between regions sensitive to self‐relevant features (Liang et al., 2021; Sui et al., 2013; Yin et al., 2021). There is also evidence that besides the MPFC and adjacent areas, processing of self‐relevant information is associated with activity in lateral posterior areas, such as the inferior parietal lobule (IPL) (van der Meer et al., 2010), posterior cingulate cortex (PCC), bilateral angular gyrus (Yaoi et al., 2015) and anterior insular cortex (ACC) (Molnar‐Szakacs & Uddin, 2013; Perini et al., 2018).

The profusion of findings indicates that the neural substrates of self‐relatedness engage broad brain regions. However, understanding the connectivity of these regions and the critical principles underlying brain responses across studies relating the processing of self‐relatedness to brain activity is a challenging task for two reasons. First, fMRI experiments on self‐relatedness are often crafted to single out a specific psychological process (e.g. evaluating personality traits and social comparison), and the correspondence across different experiments is largely unknown. Second, most studies used standard brain‐mapping analyses that enable conclusions on the involvement of specific brain regions in a task or stimuli processing. Still, the magnitude of the signal does not necessarily correlate with the importance of the respective region for the task of interest and cannot be standardized to quantify differences between brain regions (Huber, 2009). Additionally, whereas several studies have shown that task information representations are distributed throughout the brain, such studies have yet to reveal how these distributed representations are coordinated and how other brain regions use information in any one brain region to produce cognitive computations (Ito et al., 2017).

A recently emerged approach, which conceptualizes the brain as a complex, hierarchical network of functionally connected regions, has offered a new perspective in studying the neural substrates of self‐relatedness (Bressler & Menon, 2010 for review). Using this network approach, studies have consistently reported that processing of self‐relevant information is associated with a set of corresponding regions, including the MPFC and posteromedial cortices, which activity has also been observed in the absence of a specific task or stimulus during a resting state (Northoff & Bermpohl, 2004). This finding led to the suggestion that the resting‐state networks, particularly the default mode network (DMN), might be particularly implicated in supporting self‐referential processes (Qin & Northoff, 2011; Whitfield‐Gabrieli et al., 2011 for review). It was also proposed that the interaction between resting state and self‐relatedness is not limited to the DMN but may be linked to their balance to other networks such as central executive network (CEN) and posterior parietal cortex (PPC) and sensorimotor network (Northoff, 2016b).

The idea that interaction between the DMN and other brain networks may serve as a substrate for maintaining self‐referential processing opens an interesting perspective. However, although it is generally accepted that self‐referential processes are prominent at rest, the involvement of other resting‐state networks and their interaction remain to be characterized. In particular, recent evidence indicated that there are at least three other networks involved in the SPE: (i) right frontoparietal network (rFPN), which is thought to be vital for generating self‐awareness (Uddin et al., 2007); (ii) salience network (SN) contributing to self‐awareness, subjective salience of stimuli and attention allocation towards intrinsically relevant information (Uddin, 2015; Uddin et al., 2017); and (iii) cognitive control network (CCN), which is necessary when tasks require both internally and externally directed attention, as active self‐ or other‐referential tasks do (Finlayson‐Short et al., 2020). In line with this, a recent review proposed a neural framework defining the key networks supporting information flow for self‐referential processing (Sui & Gu, 2017). According to this framework, self‐referential processing is supported by the interaction between the ‘core self’ where the functional gradient between ventromedial and dorsomedial prefrontal cortices (vmPFC and dmPFC) is linked to self‐other‐related judgements and cognitive control that regulate the processing flow in bottom‐up and top‐down manner contributes to a form of ‘social saliency’ in the presence of self‐related stimuli. The interaction between the ‘core self’ and the SN, particularly between the vmPFC and insular cortex, has been associated with the magnitude of self‐biases in perception and memory (Sui & Gu, 2017).

Taken together, the work mentioned above points towards multiple networks involved in the generation of the SPE. However, this assumption has not been tested directly yet. As such, some important questions remain unanswered. Is the SPE, for instance, generated by interactions between several networks or supported by the DMN only? If the former is true, which networks are crucial for the SPE, and what is the nature of interactions between them? Furthermore, although the link between self‐referential effects and DMN received extensive empirical investigations, it is still unclear which part of the DMN contributes to the ‘core self’. For example, some studies suggested that the vmPFC is a self‐representation hub related to the functions of self‐anchor in decision‐making, self‐binding and representing the personal value of self‐related information (D'Argembeau, 2013; Sui et al., 2013; Sui & Humphreys, 2017; Wagner et al., 2012 for review). In contrast, other studies endorsed a tripartite core‐self model (MPFC, PCC, left IPL) in which self‐relatedness is thought to be driven via PCC as a region coordinating mental representation and exerting its influence on MPFC and IPL (Davey et al., 2016) via its unique anatomical position as a brain‐wide connectivity hub (Tomasi & Volkow, 2011).

In the present study, we aim to shed light on these questions using a novel approach in which neuroimaging data of the human brain are modelled as a set of networks. The underlying assumption of this approach is that neural responses to a stimulus or task are associated with changes in neural activity in some areas of the brain and a global reorganization of connectivity patterns (Bressler & Menon, 2010). A recent line of research demonstrated that cognitive performance relies on the coordination of large‐scale networks of brain regions that show highly correlated spontaneous activity during a task‐free state (Cole et al., 2014, 2016; Ito et al., 2017; Kieliba et al., 2019). It was suggested that the functional network architecture identified using resting‐state functional connectivity (FC) could plausibly reflect the routes by which activity flows during cognitive task performance (Cole et al., 2014; Smith et al., 2009; Thomason et al., 2008; van den Heuvel et al., 2009). Following these findings, exploring the cognitive relevance of task‐relevant neural topology in self‐referential processing may provide new insights into information flow across the brain and underlying group structure in large‐scale networks to shape the SPE.

Our primary hypothesis is motivated by the proposition that interactions between the ventromedial part of the DMN, cognitive control and saliency networks support the processing of self‐relevant information (Sui & Gu, 2017). On the other hand, the SPE may emerge from an interaction between parts of the DMN such as PCC, MPFC and IPL. The plausibility of this hypothesis is determined by fMRI evidence of the involvement of these areas in self‐related processes and their broader associations with a goal‐directed behaviour (Davey et al., 2016; Tomasi & Volkow, 2011). We tested these hypotheses by examining the changes in large‐scale brain networks for self versus others using a network‐based statistics (NBS) approach. The NBS is a validated statistical method to assess the whole set of interactions of brain networks by identifying topological clusters among the set of all connections (Fornito et al., 2015; Zalesky et al., 2010; Zhu et al., 2021). Importantly, in NBS, the most basic equivalent of a cluster is a connected graph component sounds to represent any putative experimental effect. A component's presence can be considered as evidence of an interconnected configuration of networks rather than being confined to a single connection or distributed over several connections that are in isolation of each other. Therefore, identifying a component including the frontal part of the DMN, cognitive control and SN for shapes associated with self compared with shapes associated with others would provide support for our primary hypothesis.

An interesting question then would be whether self‐prioritization is associated with a unique component of interconnected networks or can be explained mainly by related effects such as emotion prioritization. Evidence from behavioural, electrophysiological and imaging studies demonstrated that people prioritize emotionally valences information compared with emotionally neutral and the emotion‐prioritization effects are compatible with those generated by self‐relatedness. For example, both of them can generate robust facilitation effects on visual attention selection (Fields & Kuperberg, 2016), perceptual learning (McIvor et al., 2021) and carryover effects (Wang et al., 2016). Based on this evidence, it is not surprising that many neuroimaging studies reported neural overlap between self‐referential and emotion processing in the MPFC, ACC and PCC (Gutchess & Kensinger, 2018; Northoff et al., 2009). However, whether self and emotion processing shares some neural substrates is under continuing debate (Daley et al., 2020; Oosterwijk et al., 2017). We aim to contribute to the debate by identifying whether the brain forms the same components of interconnected networks for prioritizing self (controlling for emotion) and emotion‐relevant information.

## 2 METHOD

### 2.1 Data sets and tasks

We employed fMRI data sets from a previously reported study where healthy young adults performed two associative matching tasks using personal and emotion associations (Yankouskaya & Sui, 2021, Study 1). In the personal task, participants learned associations between simple geometrical shapes (e.g. square, circle and triangle) and personal labels (e.g. square—you; circle—friend; triangle—stranger). After learning these associations, they performed ‘shape‐label’ matching, indicating whether a presented shape‐label pair matched or mismatched associations learned earlier. The procedure for the emotion task was identical, differing only in stimuli (schematic emotional expressions depicting sadness, happiness and neutral) and different geometrical shapes (e.g. diamond, pentagon and rectangle). To validate our primary hypothesis, we used a separate data set reported in Yankouskaya, Humphreys, et al. (2017) (Study 2) where participants performed the personal task with two‐item associations (e.g. squire—you; triangle—friend).

Procedures, stimuli and stimuli presentation were identical in Study 1 and Study 2. Geometric shapes (circle, hexagon, square, rectangle, diamond and triangle) were randomly assigned to conditions in each task. The stimulus display contained a fixation cross (0.8° × 0.8°) at the centre of the screen with a shape (3.8° × 3.8°) and a label on either side of fixation. The distance between shape and label was 10°. Presentations of the shapes and labels were counterbalanced across trials. Each trial started with a fixation cross for 200 ms, followed by the stimulus display for 100 ms and a blank interval that remained for 1000 ms. Trials were separated by a jittered interstimulus interval (ranging between 2000 and 6000 ms). In each study, before entering the scanner, participants performed a short practice with the task (12 trials per task). Feedback on accuracy (words ‘Correct!’ and ‘Incorrect!’) and overall response time was provided after each trial during the practice.

Imaging data acquisition for each data set and behavioural performance are summarized in Table S1. Both studies were approved by the Central University of Oxford Research Ethics Committee (CUREC). All participants provided written informed consent.

### 2.2 fMRI data preprocessing

Raw data from both studies were preprocessed and analysed separately using SPM12 (Wellcome Trust Centre for Neuroimaging, London, UK; www.fil.ion.ucl.ac.uk/spm) running in Matlab R2020b (MathWorks, Inc., Natick, MA, USA). The preprocessing steps included slice timing correction, functional realignment and unwarp, segmentation and normalization. First, all scans were corrected for differences in slice acquisition times to make the data on each slice correspond to the same point in time. Next, slice timing correction was performed using the slice acquired at the middle of the TR as reference. Then the data were aligned across and within functional sessions and unwarped (estimation and removal of movement‐by‐susceptibility induced variance in fMRI time series). This routine realigns a time series of images acquired from the same subject using a least squares approach and a six‐parameter (rigid body) spatial transformation. Structural data were registered to the first functional frame and spatially normalized to Montreal Neurological Institute (MNI) space using SPM12 unified segmentation–normalization algorithm (Ashburner & Friston, 2005). Finally, functional data were resampled to a 91 × 109 × 91 bounding box with 2‐mm isotropic voxels. No additional spatial smoothing was applied in order to minimize artificial local spatial correlations in the whole‐brain analysis.

After the initial preprocessing in SPM12, each of the data were submitted separately to the CONN toolbox (Version 20a) for additional denoising steps and FC analyses. First, we used the ART procedure implemented in CONN for artefact detection. The results of gross head movement detection indicated that our sample did not contain participants with a head displacement exceeding 3 mm in more than 5% of volumes in any sessions. It has been suggested that FC can also be influenced by small volume‐to‐volume ‘micro’ head movements (Van Dijk et al., 2012). To ensure that micro‐head movement artefacts did not contaminate our findings, functional data with frame‐to‐frame displacements greater than 0.40 mm were censored (Power et al., 2014).

Recent studies showed that FC results can be severely affected by physiological noise (Birn et al., 2014). To address this issue, we used an anatomical component‐based noise correction method (aCompCor; Behzadi et al., 2007) that derives potential physiological and movement effects on the BOLD time series by evaluating the signal within white matter and cerebrospinal fluid (CSF) areas. It was suggested that this method does not suffer severely from systematic introduction of negative correlation (Murphy et al., 2009) while retaining some of the advantages of global signal regression (GSR) (Chai et al., 2012). The principal components of the signal from eroded white matter and CSF masks were regressed out. In the main text, we reported the results without GSR. The reason behind this decision was that our analysis focuses on the interactions across the whole set of brain networks and therefore preserving global fluctuations across these networks would be beneficial for capturing the interactions (Scalabrini et al., 2020). However, due to the ongoing controversy associated with GSR (Caballero‐Gaudes & Reynolds, 2017), we also report key findings with GSR in Table S2. The noise components from white matter and CSF, estimated subject‐motion parameters (three rotation, three translation parameters plus their associated first‐order derivatives) and outlier scans were regressed out as potential confounding effects. We also included session and task effects as additional noise components to reduce the influence of slow trends and constant task‐induced responses in the BOLD signal. Finally, a high‐pass filter (e.g. *[0.008 inf]*, which implements the standard 128‐s high‐pass used in SPM for regular task analyses) as an acceptable compromise between minimizing cross‐talk/spillage of the BOLD signal between session/conditions while still benefiting from the increased signal‐to‐noise ratio (SNR) afforded by filtering was applied to functional data.

For quality assurance, we evaluated denoising outputs for each participant and each functional run using quality‐control functional connectivity (QC‐FC) method (Ciric et al., 2017). This method computes FC between randomly selected pairs of points within the brain and evaluates whether these connectivity values are correlated with other QC measures such as subject‐motion parameters. Distributions of between‐subject correlations between FC values and QC measures after denoising indicated lack of noticeable QC‐FC associations in both data sets.

### 2.3 Network analysis

After the preprocessing and denoising steps, the residual time series from each session/task within each study were concatenated to form a condition‐specific time series of interest, in each brain region. For the first‐level analysis, we used region‐of‐interest to region‐of‐interest (ROI‐to‐ROI) connectivity (RRC) measures of large‐scale networks. The large‐scale networks ROIs were defined from default CONN's networks atlas (derived from ICA analyses based on the Human Connectome Project [HCP] data set of 497 subjects). The networks atlas delineates an extended set of classical networks: DMN (four ROIs), SensoriMotor (two ROIs), Visual (four ROIs), Salience/Cingulo‐Opercular (seven ROIs), DorsalAttention (four ROIs), FrontoParietal/Central Executive (four ROIs), Language (four ROIs) and Cerebellar (two ROIs). The Cerebellar ROIs were not included as it only had partial coverage in the participants. In total, we analysed 30 ROIs. However, rather than focusing on any of these networks in isolation, we treated all ROIs as ‘nodes’ within a whole‐brain network.

To assess changes in whole‐brain connectivity between conditions, we used the NBS analysis (Zalesky et al., 2010). First, we defined condition‐specific FC strength (i.e. FC during each task/condition) by computing weighted RRC matrices using a weighted least squares linear model with temporal weights identifying each individual experimental condition. The weights were defined as a condition‐specific boxcar time series convolved with a canonical haemodynamic response function. Weighted RRC matrices of Fisher‐transformed bivariate correlation coefficients between all ROIs/nodes (30 × 30) were calculated for each task/condition/participant. These matrices were submitted to the second‐level analysis where the differences between conditions constituting self‐prioritization (self > stranger) and emotion prioritization (happy > neutral, sad > neutral) were calculated for every edge/connection using a general linear model (GLM).

The resulting statistical parametric map for each contrast was thresholded using a priori ‘height’ threshold (uncorrected *p* < .001) to construct a set of suprathreshold links among all ROIs/nodes of between‐condition differences. It has to be noted that this ‘height’ threshold is a user‐determined parameter in NBS analysis. It was suggested that sensitivity to the test statistic threshold might reveal useful information about the nature of the effect (Zalesky et al., 2010). For example, effects presented at only conservative threshold (e.g. *p* < .001) are likely to be characterized by strong, topologically focal differences. Effects presented only at relatively liberal threshold (e.g. *p* < .05) are likely to be subtle yet topologically extended. Effects presented at both thresholds combine features of topologically focal and distributed differences. Although our analysis focused on the former threshold, we also explored changes in connectivity using the lower threshold.

Next, in the set of suprathreshold links, we identified any connected components (topological clusters) and defined the size of each component as the sum of T‐squared statistics overt all connections within each component. The critical assumption inherent to the NBS here is that connections for which the null hypothesis is false are arranged in an interconnected configuration, rather than being confined to a single connection or distributed over several connections that are in isolation of each other. In other words, the presence of a component may be evidence of a non‐chance structure for which the null hypothesis can be rejected at the level of the structure as a whole, but not for any individual connection alone (Fornito et al., 2015). Finally, a FWE‐corrected *p*‐value for each component were computed using permutation testing. The basic assumption of the permutation procedure is that under the null hypothesis, random rearranging correspondence between data points and their labels does not affect the test statistics. This would not be the case if the null hypothesis were false. The labels for each tested contrast (e.g. self > stranger) were randomly rearranged for corresponding data points according to a permutation vector of integers from 1 to the total number of data points. The same permutation vector was used for every connection (830 in total) to preserve any interdependencies between connections and constrained to remain within the same participant. The size of the largest component was recorded for each permutation, yielding an empirical null distribution for the size of the largest component size. This procedure was performed 1000 times. A FWE‐corrected *p*‐value for a component of given size was then estimated as the proportion of permutations for which the largest component was of the same size or greater and, thus, representing the likelihood under the null hypothesis of finding one of more components with this or larger mass across the entire set of networks.

To characterize the properties of each component, we report ‘size’ as the number of suprathreshold connections, ‘intensity’ (mass) measures as their overall strength (i.e. sum of absolute T‐values over these suprathreshold connections) and *p*‐values associated with these measures. In addition, we provide complementary statistics for each connection such as effect size for significant components calculated by averaging the test statistic values across significant connections and dividing by the square root of the number of subjects and between‐subject variability for each connection within a component to gain more insight into contrasts of interest.

In sum, we first assessed changes in whole‐brain connectivity between conditions forming SPE and emotion‐prioritization effects through four contrasts of interest: [self > stranger], [self > friend], [happy > neutral], [sad > neutral]. Next, we refined our account of SPE and emotion‐prioritization effects, we assessed changes in whole‐brain connectivity by contrasting SPE and emotion‐prioritization effects. Finally, to validate our finding that processing of self‐related information was associated with temporal correlation across multiple neural networks, we carried out NBS analysis using separate data set (see details in Section 2.1).

## 3 RESULTS

### 3.1 SPE

#### 3.1.1 Connections between MPFC and insular/DLPFC explained self‐prioritization

**FIGURE 1:**
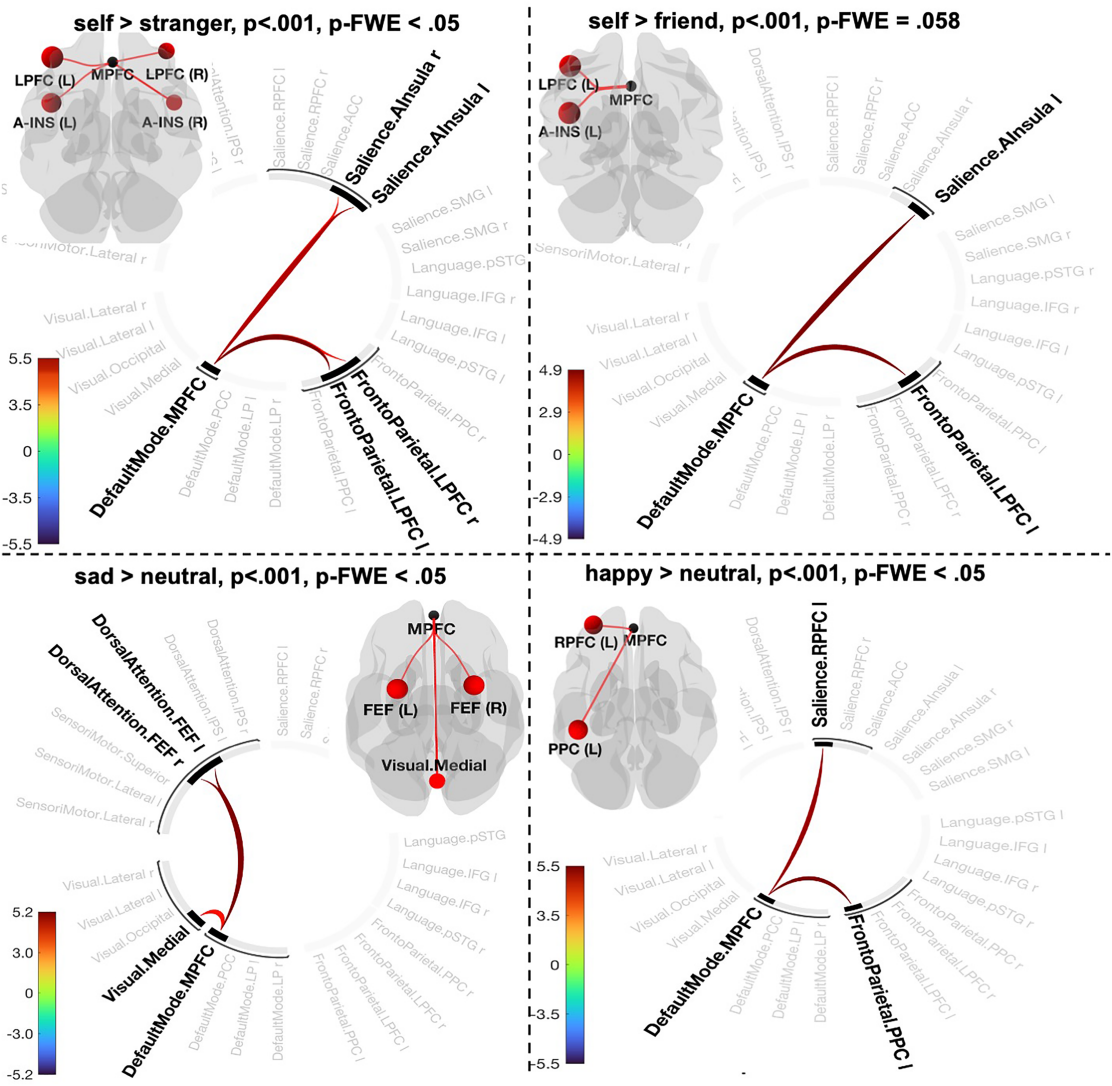
Connectogram representation of changes in pairwise network functional connectivity for contrasts [self > stranger] (a), [self > friend] (b), [sad > neutral] (c) and [happy > neutral] (d) and *p*‐statistics associated with a topological component and FWE‐corrected at network level. Glass brain visualizes spatial location of connections comprising each component. Vertical colour bars indicate *T*‐test statistics for individual connections

**FIGURE 2:**
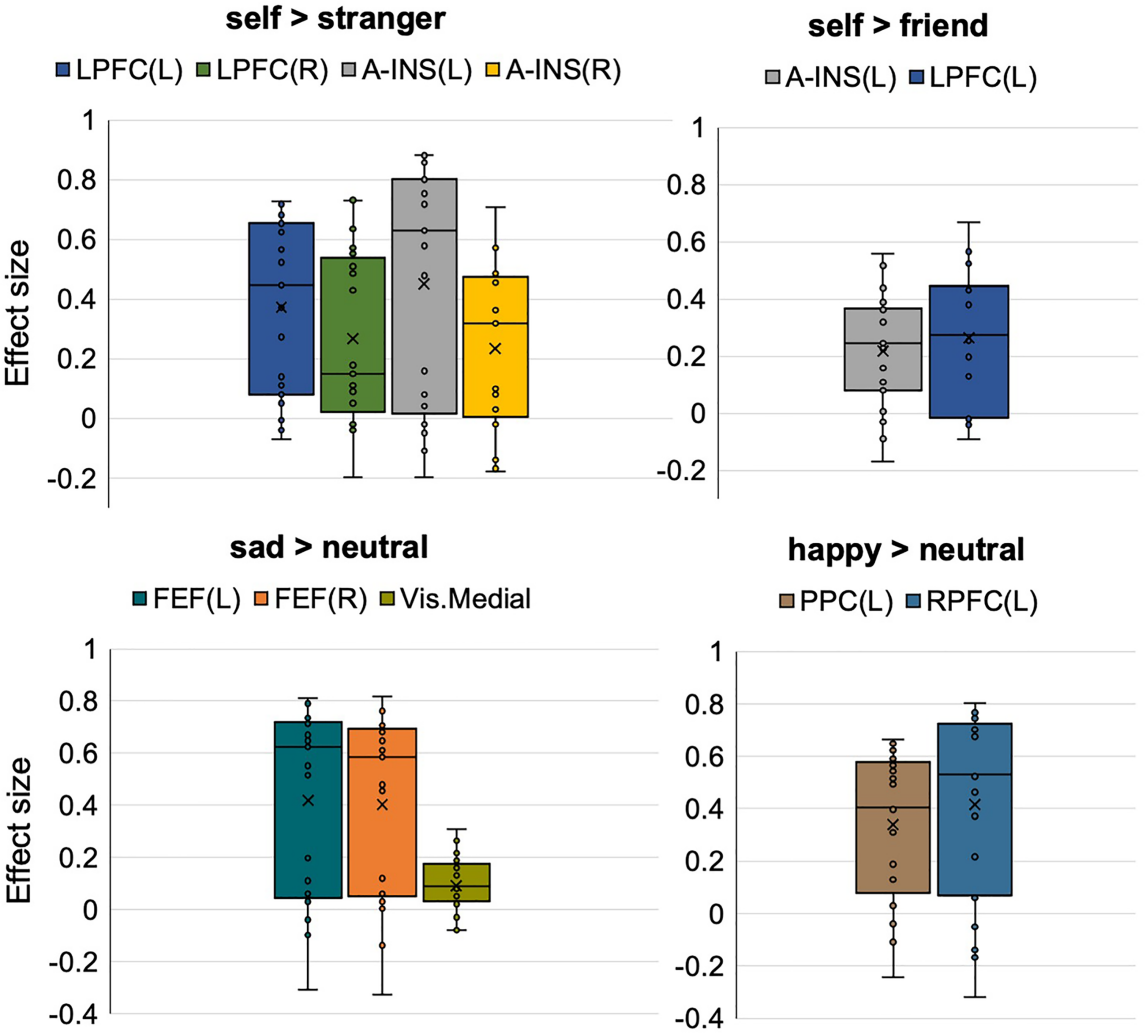
Effect sizes of individual connections (Figure 1) in contrasts defining prioritization effects in the personal task (self > stranger, self > friend) and emotion task (sad > neutral, happy > neutral). The *Y*‐axis represents Pearson correlation values where the sign indicates the direction of the effect. Individual dots correspond to subject differences in connectivity values of the conditions in each contrast. Means are denoted as X, and medians as horizontal lines within each box

Contrast [self > stranger] using *p* < .001 ‘height’ threshold revealed one topological cluster (mass = 90.64, p‐FWE = .009; size = 4; Cohen's *d* = .41, 90%CI [.29, .53]) comprising connections between the DMN (MPFC) and SN (bilateral anterior insula) and frontoparietal network (bilateral lateral prefrontal cortex) (Figure 1a). Although NBS concerns with the interconnected configuration of networks, we also extracted connectivity values for the connections comprising the component to visualize the relative contribution of each connection to the effect size of the component (Figure 2, contrast self > stranger). No significant components were found when we decreased the threshold to *p* < .05. Systematic increasing the threshold by 10% showed that the effect occurred at only conservative threshold (starting from .007 to .001) (Table S3) indicating that the contrast self > stranger is likely to be characterized by strong, topologically focal differences in FC.

Contrast [self > friend] using *p* < .001 ‘height’ threshold did not pass significance using p‐FWE threshold (observed p‐FWE value = .058). However, we report this contrast as the results are important for understanding the nature of the SPE. We found one topological cluster that resembles contrast [self > stranger] by indicating interconnection between the DMN (MPFC), frontoparietal network (left LPFC) and SN (left anterior insula) (mass = 48.99, p‐FDR = .048, p‐FWE = .058; size = 2, p‐FWE = .32) (Figure 1b; Figure 2, contrast self > friend). No significant components were found when we systematically decreased the threshold up to *p* < .05 (Table S4).

### 3.2 Positivity and negativity effects

#### 3.2.1 Connections between MPFC and FEF explained the negativity effect

Negative emotion bias (contrast [sad > neutral]) showed one significant component including the DMN (MPFC), dorsal attention network (DAN) (bilateral frontal eye fields) and visual medial network (VMN) (mass = 82.91, p‐FWE = .013; size = 4; Cohen's *d* = .37, 90%CI [.19, .51]) (Figure 1c; Figure 2, contrast sad > neutral). It has to be noted that the component size displayed in Figure 1c is determined by positive functional connectivity in both directions (DMN.MPFC to Visual. Medial and Visual. Medial to DMN.MPFC). Systematic varying the ‘height’ threshold indicated that this effect occurred only at more conservative threshold (*p* < .004–.0006) (Table S5).

#### 3.2.2 Connections between MPFC and RPFC/PPC explained the positivity effect

Positive emotion prioritization defined by contrast [happy > neutral] reveal one topological cluster comprising the MPFC of DMN network, frontoparietal network (left PPC) and SN (left rostral prefrontal cortex [RPFC]) (*p* < .001, mass = 56.50, p‐FWE = .034; size = 2; Cohen's *d* = .32, 90%CI [.15, .48]) (Figure 1d; Figure 2, contrast happy > neutral). Decreasing the ‘height’ threshold (*p* < .003) showed slightly larger component by additional connection between the DMN (MPFC) and language network (posterior superior temporal gyrus [p‐STG]) yielding in total statistics with mass = 78.93, p‐FWE = .032; size = 3; Cohen's *d* = .32, 90%CI [.15, .48]. Further decreasing of the ‘height’ threshold revealed no significant results (Table S6).

### 3.3 Differences between self and emotion prioritization effects

#### 3.3.1 Positive connections between MPFC/r‐FPN/SN and negative connections between MPFC/FEF and LP/l‐FPN explained the self‐negativity effect

**FIGURE 3:**
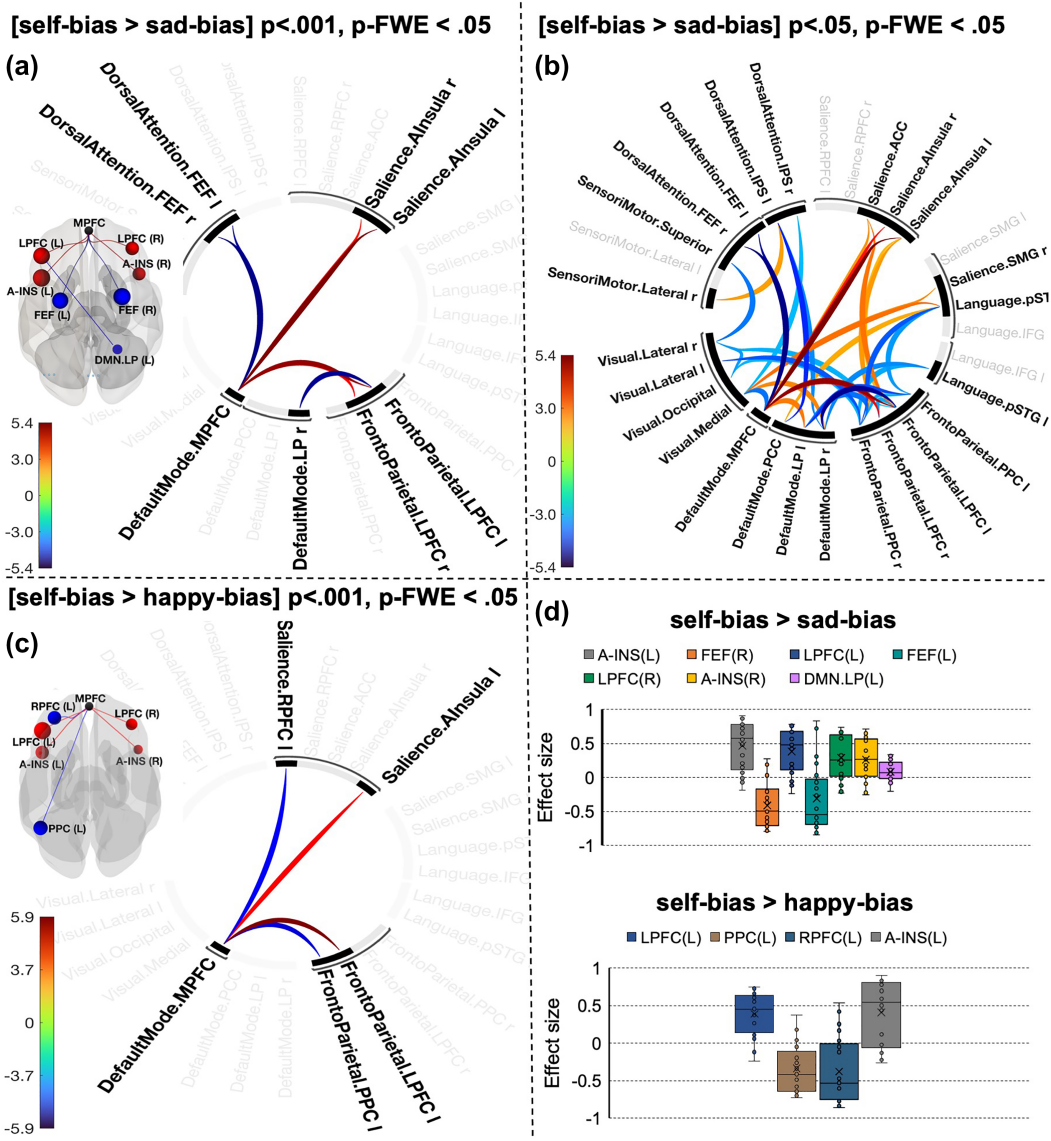
Connectogram representation of changes in pairwise network functional connectivity for contrasts [self‐bias > sad‐bias] (a,b) and [self‐bias > happy‐bias] (c). Self‐bias was defined as contrast [self > stranger]; happy‐bias and sad‐bias were defined by contrasting happy and sad emotions with a neutral expression. Glass brain visualizes the spatial location of connections. Vertical colour bars indicate *T*‐test statistics for individual connections. (d) The effect size of each connection within the components

Contrasting self‐ and sad‐prioritization effects (defined as [self > stranger] − [sad > neutral]) revealed a large component comprising eight connections between the DMN, SN (bilateral anterior insula), frontoparietal network (bilateral lateral prefrontal cortex) and DAN (bilateral frontal eye field) (‘height’ threshold *p* < .001; mass = 198.91, p‐FWE < .001, size = 8; Cohen's *d* = .37 90% CI [.20, .51]). Furthermore, the NBS indicated that the difference between self‐ and sad‐prioritization effects is determined by interplay between DMN and left frontoparietal and bilateral DAN (negative correlation) and positive correlations between the medial part of the DMN, SN and rFPN (Figure 3a). Interestingly, applying a lower cluster‐forming threshold (*p* < .05) identified a large and spatially extended component comprising 66 connections (mass = 615.64, size = 66, p‐FWE = .024) (Figure 3b). Gradual increasing of the threshold (up to *p* < .008) supported the identification of this component but spatially restricted (Table S7). As this component presents across a range of threshold, it is likely to be characterized by a combination of both subtle yet topologically extended differences and strong but topologically focal differences.

#### 3.3.2 Positive connections between MPFC/SN/PPC/DLPFC explained the self‐positivity effect

The differences between self‐ and positive‐emotion biases defined by the contrast [[self > stranger] − [happy > neutral]] at conservative thresholds (*p* < .001–.003) yielded in a component comprising connections between DMN (MPFC) and SN (left anterior insula and left rostral prefrontal cortex) and frontoparietal network (left PCC and left lateral prefrontal cortex) (mass = 99.21, size = 4, p‐FWE = .007; Cohen's *d* = .49, 90%CI [.42, .55]). This effect was diminished at more liberal thresholds indicating strong, topologically focal differences (Table S8).

### 3.4 Validation of the topological cluster for self‐prioritization

**FIGURE 4:**
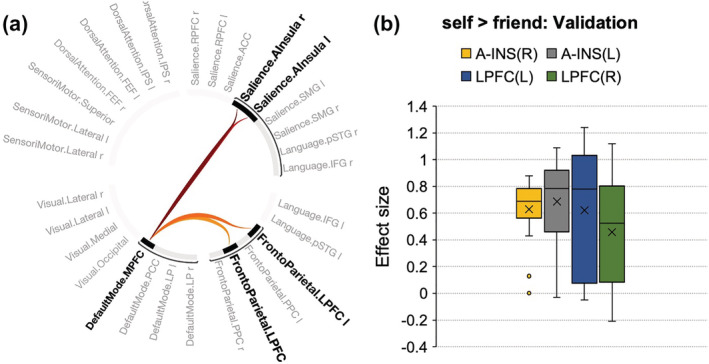
(a) Connectogram representation of changes in pairwise network functional connectivity for contrasts [self > friend] in separate data set (p‐FWE < .001). (b) The effect size of each connection within the components

Similar to contrast [self > stranger] in the former data set, contrast [self > friend] using *p* < .001 ‘height’ threshold revealed one topological cluster (mass = 220.92, p‐FWE < .001; size = 4; Cohen's *d* = .60, 90%CI [.23, .96]) comprising connections between the DMN (MPFC) and SN (bilateral anterior insula) and frontoparietal network (bilateral lateral prefrontal cortex) (Figure 4a,b). Systematic increasing of the threshold by 10% showed that the effect occurred at both more liberal threshold (.02) and conservative threshold (starting from .009 to .00006) (Table S9).

## 4 DISCUSSION

The involvement of the DMN in the processing of self‐related information is well established and often used as a synonym of self‐referential mental activity (Davey et al., 2016). However, it remains unclear whether prioritization of self‐related information solely depends on the interplay between parts of the DMN or is supported by a unique community of multiple brain networks. In the present study, we addressed this question by identifying topological clusters of networks involved in the SPE through two task‐based fMRI studies.

Previous studies examining the neural substrates of self‐referential processing used FC between brain regions during task performance (Qin & Northoff, 2011) or resting state (Sheline et al., 2010), or both (Davey et al., 2016). Although these approaches have shown undeniable merits in revealing neural correlates of self‐referential processing, they are limited in inferences of how self‐relatedness is mapped into a large‐scale functional architecture of the brain. The present study addressed this limitation by testing the changes in the intrinsic functional organization during a task that robustly generates the SPE.

### 4.1 Topological cluster for the SPE

Our results provided evidence that the processing of self‐related information was associated with temporal correlation across multiple neural networks, including the medial frontal part of the DMN (MPFC‐DMN), insular part of the salience network (AI‐SN) and lateral prefrontal cortex of the frontoparietal network (LPFC‐FPN). One important observation in the previous studies is that the part of the FPN that corresponds to the LPFC‐FPN in the present study exhibited positive correlations with the DMN across various tasks, including self‐referential processing (Crittenden et al., 2015; Dixon et al., 2018). For example, it was suggested that the LPFC‐FPN might preferentially contribute to executive control in the context of introspective processes and emotion, exerting a general constraint that keeps one's focus on task‐relevant material. In addition, the DMN plays a role in bringing conceptual–associative knowledge to bear on current thought and perception (Dixon et al., 2018). The positive coupling between these networks for self and negative coupling for non‐self (as follows from the contrast [stranger > self]) indicate some forms of cognitive control to facilitate self‐representation or suppress non‐self‐representation context in the MPFC. This interpretation aligns with recent evidence that FPN can flexibly adjust connectivity to DMN exhibiting differential coupling patterns in every task condition (Dixon et al., 2018). Interestingly, evidence from evolutionary and developmental studies suggests that the LPFC comprises some human‐specific efferent connections with the caudal part of the MPFC (Badre & D'Esposito, 2009). Exploring the strengthened links between the LPFC and the MPFC and their role in maintaining the SPE may bring new ideas on the nature of self‐prioritization.

Interactions between the DMN, FPN and insular cortex within the SN have been well documented in FC studies in healthy individuals (Finlayson‐Short et al., 2020; Modinos et al., 2009; van Buuren et al., 2020) and patients (Garrity et al., 2007). For example, it was suggested that the interplay between the DMN and anterior insula responded to the degree of subjective salience (Menon & Uddin, 2010). Furthermore, there is evidence of causal interactions between the FPN, DMN and SN where the anterior insula plays a coordinating role in switching the FPN and DMN across task paradigms and stimuli (Sridharan et al., 2008). The switching function of the SN was linked to facilitating access to attention and working memory resources when a salient event is detected and rapid access to the motor system (Menon & Uddin, 2010). The proposed mechanisms can explain behavioural results in the present study and in line with other work using the associative matching task (Desebrock et al., 2018; Schäfer et al., 2015; Wang et al., 2016; Yankouskaya, Palmer, et al., 2017).

Building upon this knowledge, our finding of a component of interacting networks (DMN, FPN and SN) suggests that the self‐related processing requires control of information processing and generating the ‘salience map’ to motivate the information processing (Shi et al., 2021). This finding is in line with a recently proposed neural model of the self as an object (Sui & Gu, 2017). However, it places some constraints on the view that self‐reference is an automatic mechanism (Soares et al., 2019). Although it is generally accepted that self‐relatedness is associated with FC within the DMN, which activity is more prominent at rest, the effortlessness of self‐referential mental processes is limited when decision‐making is required (Hugdahl et al., 2015; Vatansever et al., 2017).

The NBS indicate that the component of networks associated with the SPE emerged as a result of a strong focal effect confined to relatively few connections. Although specific characteristics of focal versus distributed network connectivity and its relation to behavioural effect remain largely unknown, evidence from fundamental neuroscience indicates that pooling together information from more neurons does not improve behavioural sensitivity (Bouton et al., 2018; Kok et al., 2012; Shadlen et al., 1996). For example, it was demonstrated that damage of these focal regions dramatically disrupted task performance, whereas distributed lesions did not impair task performance (Bouton et al., 2018). The authors suggested that focal activity is critical for cognitive processes such as perceptual decisions, whereas distributed activations across regions could reflect the reuse of sensory information for higher level operations, such as extraction of meaning. Hinging on these findings, we interpret the focal effect of connectivity between DMN, FPC and SN as critical to cognitive operations for the SPE.

### 4.2 Validation of the topological cluster for SPE

The NBS analysis using our validation data set confirmed that SPE is associated with the interaction between DMN, FPC and SN. However, in contrast to our former finding that the interaction between the three networks was confined to a strong focal effect, we observed both focal and distributed effects. The difference between these findings reflects the nature of contrasts we assessed (i.e. self > stranger in the former data set and self > friend in the validation set). Previous behavioural and fMRI studies using the shape‐label matching task consistently reported that both associations with self and associations with friend bias perception compared with associations formed with a stranger (Macrae et al., 2017; Sun et al., 2016). However, the magnitude of other‐associations depends on personal closeness to the self (Oyserman et al., 2012; Yankouskaya et al., 2020), identity relevance (Golubickis et al., 2020) and culture (Jiang et al., 2019). From this perspective, it is unsurprising that these factors could add a distributed effect to the interaction between networks. The most important finding is that the same topological component of interconnected networks was observed in both self > stranger and self > friend contrasts in separate data sets. The consistency of this finding suggests that SPE is a product of information flow between DMN, FPN and SN and these three networks' involvement is critical for generating this effect. What remains to be seen, however, is how the information flows. In particular, the DMN has been suggested as a ‘global hub’ or ‘integrator’ exerting its influence over the FPN during conscious processing of information (Hugdahl et al., 2015; Sui, 2016; Vatansever et al., 2017). In line with this notion, our findings showed increased positive connectivity between the MPFC of the DMN and two other networks (FPN and SN) for self > other. However, the topological component does not reveal temporal correlations between the FPN and SN. Although we cannot rule out that the interaction between these networks is orchestrated by the DMN, the limitations of NBS in exploring the directionality of information flow call for future research.

### 4.3 The uniqueness of topological cluster for the SPE

Our results demonstrated that the SPE could not be explained by related prioritization effects such as emotion. Instead, we found that happy emotion was associated with a distinct component formed by interactions between the left rostral prefrontal part of SN (RPFC), posterior parietal part of FPN and MPFC of DMN, while sad emotion reveals a cluster of the DMN, DAN and VMN. These findings are in line with the broad literature on organizational principles of the human brain functional connectome during the processing of affective information (Iordan & Dolcos, 2017; Sheline et al., 2010; Zhang et al., 2015). For example, it was proposed that the RPFC is coupled with the DMN and posterior parietal cortices during emotion processing in healthy individuals. However, in aberrant functional connectivity within nodes of the DMN, FPN and SN, the RPFC ‘hot‐wires’ the tree networks together, leading to various depressive symptoms (Fadel et al., 2021; Scalabrini et al., 2020; Sheline et al., 2010).

Our finding that the SPE is associated with a distinct set of interacted networks highlights two important points. One of them is theoretical and reflects the long‐standing debates about the relationship between the self and emotion processing within the more general domain of social perception (Heinzel & Northoff, 2014; Sui & Gu, 2017). A large body of research, including our previous work, reported overlapped neural substrates for emotion‐prioritization effect and SPE (Kim et al., 2016; Smith et al., 2018). In particular, the effects of positive emotion resemble those triggered by self‐relatedness (Yankouskaya & Sui, 2021). Most of the work used a seed‐to‐voxel connectivity analysis with the MPFC as a seed commonly reported in both self‐referential and emotion processing. Although this approach can provide us with valuable information about the functional network of a particular region, it cannot capture the complexity of interactions between intrinsic brain networks that may be functionally relevant. Our results demonstrated that the MPFC is involved in either positive emotion prioritization or self‐prioritization. However, the difference between these two effects reflects the interaction between the MPFC as part of the DMN and other networks such as SN and FPN. In particular, self‐prioritization is associated with positive coupling between the MPFC, SN (anterior insula) and FPN (lateral prefrontal cortex) and negative coupling between the MPFC, the rostral prefrontal cortex of the SN and the posterior parietal part of the FPN. The second point is methodological. The network‐based approach allowed us to pin down some properties of perceiving sad emotion and its relation to self that other existing techniques cannot easily capture. Previous attempts to localize areas involved in the processing of sad emotional expression provided highly inconsistent results reporting activity in the orbitofrontal areas, amygdala, insula, frontoparietal areas, ACC and MPFC (for review, see Lindquist et al., 2012; Touroutoglou et al., 2015). Our finding of a distributed component for perceiving a sad emotional expression at the lower threshold commonly reported for statistical inferences (FWE < .05) may partly explain this inconsistency. However, we also found a strong focal component where the DMN (MPFC) showed positive interaction with bilateral DAN and VMN for sad versus neutral expression. The DAN comprises areas of the ‘dorsal attention’ system, which is typically engaged in the appraisal of arousing information (Sander et al., 2018). According to recent research, emotion schemas are embedded in the visual system reflecting top‐down modulations from higher cortical areas (Kragel et al., 2019). The coupling between the DMN, DAN and VMN may indicate the mainstream of information flow for processing sad emotion and contribute to our understanding of the neural basis of its carryover effects (Qiao‐Tasserit et al., 2017).

### 4.4 Two models of ‘core‐self’ system

Recently, two neural models of ‘core‐self’ system were proposed (Davey et al., 2016; Sui & Gu, 2017). One of them includes the MPFC, PCC and left IPL as key nodes operating within the DMN (Davey et al., 2016). According to this model, self‐related processes are driven via PCC, which had a positive influence on activity in MPFC and IPL, and MPFC had a moderating influence on PCC. The coordinating role of the PCC is thought to be driven by rich anatomical and functional connections between the PCC and the rest of the brain that places this region as a good candidate to orchestrate mental representations such as self‐reference (Davey et al., 2016). The second model put forward the hypothesis that the integrative property of the self is associated with the functions of the MPFC as part of the DMN and how this region is coupled with the SN and regions involved in cognitive control such as DLPFC (Guan et al., 2021; Sui & Gu, 2017). The results of the NBS in the present study support the integrative ‘core‐self’ model (Sui & Gu, 2017) by demonstrating that the SRE is generated via interactions between the MPFC and areas outside the DMN such as LPFC and AI. Furthermore, our findings indicate that the MPFC may play the hub role in this interaction. Measures of directed influence based on multivariate fMRI time series such as conditional Granger causality analysis (Zhou et al., 2009) and transfer entropy (Ursino et al., 2020) may provide a precise estimation of the directionality and the strength of connectivity between neural populations within the component of interacting networks supporting the SPE. However, our finding that the FPN and SN are linked to the MPFC but not to each other points towards the integrative role of the MPFC that is supported by mounting evidence from the literature (D'Argembeau, 2013; Meyer & Lieberman, 2018; Northoff, 2016; Wagner et al., 2012).

It has to be noted that recent large‐scale meta‐analysis (Qin et al., 2020) indicates that the contradiction between two models of the ‘core‐self’ system can be explained if self is considered as nested hierarchically organized layers of different aspects of self such as interoceptive self, extero‐proprioceptive self and mental (cognitive) self. According to this meta‐analysis, each of the hierarchical levels of self recruits both overlapping and separate regions depending on which aspect of self is engaged. For example, embodied self (extero‐proprioceptive self), which is close to our task, recruits regions associated with the MPFC‐node of the DMN, whereas cognitive self may recruit parietal nodes of FPN. Our results of the self‐related processing (e.g. MPFC and insula) are consistent with this meta‐analysis. However, our results indicate that the MPFC rather than the insula was a connection hub for emotion‐related processing when self‐referential information was absent.

### 4.5 Limitations

The literature is highly inconsistent in the precision mapping of brain networks. This inconsistency stems from different approaches to anatomical and functional parcellations (Arslan et al., 2018; Bressler & Menon, 2010; Power et al., 2011), related controversies about isomorphism between anatomical and functional spaces (Cole et al., 2014; Eickhoff et al., 2018; Petersen & Sporns, 2015) and the lack of consistent naming conventions and the number of large‐scale networks (Uddin et al., 2019). The substantial disparity in parcellation scales and nomenclature across different studies limits comparisons between our study and previous work.

## 5 CONCLUSION

We found that the processing of self‐related information is a product of information flow between DMN (MPFC as a hub), FPN and SN, suggesting that the SPE requires control of information flow and generating the ‘salience map’ to motivate the information processing. Our findings indicate that the MPFC may play the hub role in orchestrating interactions between these networks. The SPE could not be explained by related effects such as prioritization of positive or negative emotions. We found that happy emotion was associated with a distinct component formed by interactions between the left rostral prefrontal part of SN, posterior parietal part of FPN and MPFC, whereas processing of sad emotion formed a cluster of the DMN, DAN and VMN. These findings contribute to theoretical debates about the relationship between the self and emotion processing within the more general domain of social cognition and mood disorders.

## CONFLICT OF INTEREST

The authors have indicated they have no potential conflicts of interest to disclose.

## ETHICS STATEMENT

The study was conducted according to the guidelines of the Declaration of Helsinki, and approved by the Central University Research Ethics Committee (CUREC) of the University of Oxford (protocol code for both studies MSD‐IDEC‐C1‐2013‐183, 01.06.2013).

## AUTHOR CONTRIBUTIONS

Both authors were involved in the design and conceptualization of the study. A.Y. was the main writer of the manuscript. J.S. contributed to the discussion of the results and editing. All authors have read and agreed to the published version of the manuscript.

### PEER REVIEW

The peer review history for this article is available at https://publons.com/publon/10.1111/ejn.15612.

## Supporting information


**Table S1.** Summary of datasets and behavioural results.
**Table S2.** Comparison key findings* with** and without Global Signal regression (GSR). All contrasts were defined using ‘height’ threshold of p < 0.001 (uncorrected) and cluster‐corrected p‐FWE < 0.05
**Table S3.** The results of Network Based Statistic analysis with varied ‘height’ thresholds for contrast [***self > stranger***] (p‐value for each component was FWE corrected). NS denotes that a component did not survive the FWE correction.
**Table S4.** The results of Network Based Statistic analysis with varied ‘height’ thresholds for contrast [***self > friend***] (p‐value for each component was FWE corrected). NS denotes that a component did not survive the FWE correction.
**Table S5.** The results of Network Based Statistic analysis with varied ‘height’ thresholds for contrast [***sad > neutral***] (p‐value for each component was FWE corrected). NS denotes that a component did not survive the FWE correction.
**Table S6.** The results of Network Based Statistic analysis with varied ‘height’ thresholds for contrast [***happy > neutral***] (p‐value for each component was FWE corrected). NS denotes that a component did not survive the FWE correction.
**Table S7.** The results of Network Based Statistic analysis with varied ‘height’ thresholds for contrast [***self‐bias > sad‐bias***] (p‐value for each component was FWE corrected). NS denotes that a component did not survive the FWE correction.
**Table S8.** The results of Network Based Statistic analysis with varied ‘height’ thresholds for contrast [***self‐bias > happy‐bias***] (p‐value for each component was FWE corrected). NS denotes that a component did not survive the FWE correction.
**Table S9.** The results of Network Based Statistic analysis with varied ‘height’ thresholds for contrast [***self > friend***] in validation data set (p‐value for each component was FWE corrected). NS denotes that a component did not survive the FWE correction.

## Data Availability

Available on request to the corresponding author.

## References

[ejn15612-bib-0001] Arslan, S. , Ktena, S. I. , Makropoulos, A. , Robinson, E. C. , Rueckert, D. , & Parisot, S. (2018). Human brain mapping: A systematic comparison of parcellation methods for the human cerebral cortex. NeuroImage, 170, 5–30. 10.1016/j.neuroimage.2017.04.014 28412442

[ejn15612-bib-0002] Ashburner, J. , & Friston, K. J. (2005). Unified segmentation. NeuroImage, 26(3), 839–851. 10.1016/j.neuroimage.2005.02.018 15955494

[ejn15612-bib-0003] Badre, D. , & D'Esposito, M. (2009). Is the rostro‐caudal axis of the frontal lobe hierarchical? Nature Reviews. Neuroscience, 10(9), 659–669. 10.1038/nrn2667 19672274 PMC3258028

[ejn15612-bib-0004] Behzadi, Y. , Restom, K. , Liau, J. , & Liu, T. T. (2007). A component based noise correction method (CompCor) for BOLD and perfusion based fMRI. NeuroImage, 37(1), 90–101. 10.1016/j.neuroimage.2007.04.042 17560126 PMC2214855

[ejn15612-bib-0005] Birn, R. M. , Cornejo, M. D. , Molloy, E. K. , Patriat, R. , Meier, T. B. , Kirk, G. R. , Nair, V. A. , Meyerand, M. E. , & Prabhakaran, V. (2014). The influence of physiological noise correction on test‐retest reliability of resting‐state functional connectivity. Brain Connectivity, 4(7), 511–522. 10.1089/brain.2014.0284 25112809 PMC4146390

[ejn15612-bib-0006] Bouton, S. , Chambon, V. , Tyrand, R. , Guggisberg, A. G. , Seeck, M. , Karkar, S. , van de Ville, D. , & Giraud, A. L. (2018). Focal versus distributed temporal cortex activity for speech sound category assignment. Proceedings of the National Academy of Sciences of the United States of America, 115(6), E1299–E1308. 10.1073/pnas.1714279115 29363598 PMC5819402

[ejn15612-bib-0007] Bressler, S. L. , & Menon, V. (2010). Large‐scale brain networks in cognition: Emerging methods and principles. Trends in Cognitive Sciences, 14(6), 277–290. 10.1016/j.tics.2010.04.004 20493761

[ejn15612-bib-0008] Caballero‐Gaudes, C. , & Reynolds, R. C. (2017). Methods for cleaning the BOLD fMRI signal. NeuroImage, 154, 128–149. 10.1016/j.neuroimage.2016.12.018 27956209 PMC5466511

[ejn15612-bib-0009] Chai, X. J. , Castañón, A. N. , Ongür, D. , & Whitfield‐Gabrieli, S. (2012). Anticorrelations in resting state networks without global signal regression. NeuroImage, 59(2), 1420–1428. 10.1016/j.neuroimage.2011.08.048 21889994 PMC3230748

[ejn15612-bib-0010] Ciric, R. , Wolf, D. H. , Power, J. D. , Roalf, D. R. , Baum, G. L. , Ruparel, K. , Shinohara, R. T. , Elliott, M. A. , Eickhoff, S. B. , Davatzikos, C. , Gur, R. C. , Gur, R. E. , Bassett, D. S. , & Satterthwaite, T. D. (2017). Benchmarking of participant‐level confound regression strategies for the control of motion artifact in studies of functional connectivity. NeuroImage, 154, 174–187. 10.1016/j.neuroimage.2017.03.020 28302591 PMC5483393

[ejn15612-bib-0011] Cole, M. W. , Bassett, D. S. , Power, J. D. , Braver, T. S. , & Petersen, S. E. (2014). Intrinsic and task‐evoked network architectures of the human brain. Neuron, 83(1), 238–251. 10.1016/j.neuron.2014.05.014 24991964 PMC4082806

[ejn15612-bib-0012] Cole, M. W. , Ito, T. , Bassett, D. S. , & Schultz, D. H. (2016). Activity flow over resting‐state networks shapes cognitive task activations. Nature Neuroscience, 19(12), 1718–1726. 10.1038/nn.4406 27723746 PMC5127712

[ejn15612-bib-0013] Crittenden, B. M. , Mitchell, D. J. , & Duncan, J. (2015). Recruitment of the default mode network during a demanding act of executive control. eLife, 4, e06481. 10.7554/eLife.06481 25866927 PMC4427863

[ejn15612-bib-0014] Cunningham, S. J. , & Turk, D. J. (2017). Editorial: A review of self‐processing biases in cognition. Quarterly Journal of Experimental Psychology (2006), 70(6), 987–995. 10.1080/17470218.2016.1276609 28059625

[ejn15612-bib-0015] Daley, R. T. , Bowen, H. J. , Fields, E. C. , Parisi, K. R. , Gutchess, A. , & Kensinger, E. A. (2020). Neural mechanisms supporting emotional and self‐referential information processing and encoding in older and younger adults. Social Cognitive and Affective Neuroscience, 15(4), 405–421. 10.1093/scan/nsaa052 32301982 PMC8561439

[ejn15612-bib-0016] D'Argembeau, A. (2013). On the role of the ventromedial prefrontal cortex in self‐processing: The valuation hypothesis. Frontiers in Human Neuroscience, 7, 372. 10.3389/fnhum.2013.00372 23847521 PMC3707083

[ejn15612-bib-0017] Davey, C. G. , Pujol, J. , & Harrison, B. J. (2016). Mapping the self in the brain's default mode network. NeuroImage, 132, 390–397. 10.1016/j.neuroimage.2016.02.022 26892855

[ejn15612-bib-0018] Desebrock, C. , Sui, J. , & Spence, C. (2018). Self‐reference in action: A speed‐accuracy advantage in response to newly self‐associated stimuli pervades rapid‐aiming arm movements on a perceptual‐matching task. Acta Psychologica, 190, 258–266. 10.1016/j.actpsy.2018.08.009 30153556

[ejn15612-bib-0019] Dixon, M. L. , de la Vega, A. , Mills, C. , Andrews‐Hanna, J. , Spreng, R. N. , Cole, M. W. , & Christoff, K. (2018). Heterogeneity within the frontoparietal control network and its relationship to the default and dorsal attention networks. Proceedings of the National Academy of Sciences of the United States of America, 115(7), E1598–E1607. 10.1073/pnas.1715766115 29382744 PMC5816169

[ejn15612-bib-0020] Eickhoff, S. B. , Constable, R. T. , & Yeo, B. (2018). Topographic organization of the cerebral cortex and brain cartography. NeuroImage, 170, 332–347. 10.1016/j.neuroimage.2017.02.018 28219775 PMC5563483

[ejn15612-bib-0021] Fadel, E. , Boeker, H. , Gaertner, M. , Richter, A. , Kleim, B. , Seifritz, E. , Grimm, S. , & Wade‐Bohleber, L. M. (2021). Differential alterations in resting state functional connectivity associated with depressive symptoms and early life adversity. Brain Sciences, 11(5), 591. 10.3390/brainsci11050591 34063232 PMC8147478

[ejn15612-bib-0022] Fields, E. C. , & Kuperberg, G. R. (2016). Dynamic effects of self‐relevance and task on the neural processing of emotional words in context. Frontiers in Psychology, 6, 2003. 10.3389/fpsyg.2015.02003 26793138 PMC4710753

[ejn15612-bib-0023] Finlayson‐Short, L. , Davey, C. , & Harrison, B. (2020). Neural correlates of integrated self and social processing. Social Cognitive and Affective Neuroscience, 15(9), 941–949. 10.1093/scan/nsaa121 32901818 PMC7647375

[ejn15612-bib-0024] Fornito, A. , Zalesky, A. , & Breakspear, M. (2015). The connectomics of brain disorders. Nature Reviews. Neuroscience, 16(3), 159–172. 10.1038/nrn3901 25697159

[ejn15612-bib-0025] Frewen, P. , Schroeter, M. L. , Riva, G. , Cipresso, P. , Fairfield, B. , Padulo, C. , Kemp, A. H. , Palaniyappan, L. , Owolabi, M. , Kusi‐Mensah, K. , Polyakova, M. , Fehertoi, N. , D'Andrea, W. , Lowe, L. , & Northoff, G. (2020). Neuroimaging the consciousness of self: Review, and conceptual‐methodological framework. Neuroscience and Biobehavioral Reviews, 112, 164–212. 10.1016/j.neubiorev.2020.01.023 31996300

[ejn15612-bib-0026] Garrity, A. G. , Pearlson, G. D. , McKiernan, K. , Lloyd, D. , Kiehl, K. A. , & Calhoun, V. D. (2007). Aberrant "default mode" functional connectivity in schizophrenia. The American Journal of Psychiatry, 164(3), 450–457. 10.1176/ajp.2007.164.3.450 17329470

[ejn15612-bib-0027] Golubickis, M. , Falbén, J. K. , Ho, N. , Sui, J. , Cunningham, W. A. , & Neil Macrae, C. (2020). Parts of me: Identity‐relevance moderates self‐prioritization. Consciousness and Cognition, 77, 102848. 10.1016/j.concog.2019.102848 31731031

[ejn15612-bib-0028] Golubickis, M. , Falben, J. K. , Sahraie, A. , Visokomogilski, A. , Cunningham, W. A. , Sui, J. , & Macrae, C. N. (2017). Self‐prioritization and perceptual matching: The effects of temporal construal. Memory & Cognition, 45(7), 1223–1239. 10.3758/s13421-017-0722-3 28593461 PMC5605582

[ejn15612-bib-0029] Guan, F. , Liu, G. , Pedersen, W. S. , Chen, O. , Zhao, S. , Sui, J. , & Peng, K. (2021). Neurostructural correlation of dispositional self‐compassion. Neuropsychologia, 160, 107978. 10.1016/j.neuropsychologia.2021.107978 34339716

[ejn15612-bib-0030] Gutchess, A. , & Kensinger, E. A. (2018). Shared mechanisms may support mnemonic benefits from self‐referencing and emotion. Trends in Cognitive Sciences, 22(8), 712–724. 10.1016/j.tics.2018.05.001 29886010 PMC6652178

[ejn15612-bib-0031] Heinzel, A. , & Northoff, G. (2014). The relationship of self‐relatedness and emotional processing. Journal of Consciousness Studies, 21(9–10), 30–48. https://www.ingentaconnect.com/content/imp/jcs/2014/00000021/F0020009/art00002

[ejn15612-bib-0032] Huber, C. G. (2009). Interdependence of theoretical concepts and neuroimaging data. Poiesis & Praxis, 6, 203–217. 10.1007/s10202-009-0069-3

[ejn15612-bib-0033] Hugdahl, K. , Raichle, M. E. , Mitra, A. , & Specht, K. (2015). On the existence of a generalized non‐specific task‐dependent network. Frontiers in Human Neuroscience, 9, 430. 10.3389/fnhum.2015.00430 26300757 PMC4526816

[ejn15612-bib-0034] Iordan, A. D. , & Dolcos, F. (2017). Brain activity and network interactions linked to valence‐related differences in the impact of emotional distraction. Cerebral Cortex (New York, N.Y.: 1991), 27(1), 731–749. 10.1093/cercor/bhv242 26543041

[ejn15612-bib-0035] Ito, T. , Kulkarni, K. , Schultz, D. , Mill, R. , Chen, R. , Solomyak, L. , & Cole, M. (2017). Cognitive task information is transferred between brain regions via resting‐state network topology. Nature Communications, 8, 1027. 10.1038/s41467-017-01000-w PMC5715061 29044112

[ejn15612-bib-0036] Jiang, M. , Wong, S. K. M. , Chung, H. K. S. , Sun, Y. , Hsiao, J. H. , Sui, J. , & Humphreys, G. W. (2019). Cultural orientation of self‐bias in perceptual matching. Frontiers in Psychology, 10, 1469. 10.3389/fpsyg.2019.01469 31316430 PMC6610885

[ejn15612-bib-0037] Kieliba, P. , Madugula, S. , Filippini, N. , Duff, E. P. , & Makin, T. R. (2019). Large‐scale intrinsic connectivity is consistent across varying task demands. PLoS ONE, 14(4), e0213861. 10.1371/journal.pone.0213861 30970031 PMC6457563

[ejn15612-bib-0038] Kim, E. J. , Kyeong, S. , Cho, S. W. , Chun, J. W. , Park, H. J. , Kim, J. , Kim, J. , Dolan, R. J. , & Kim, J. J. (2016). Happier people show greater neural connectivity during negative self‐referential processing. PLoS ONE, 11(2), e0149554. 10.1371/journal.pone.0149554 26900857 PMC4763307

[ejn15612-bib-0039] Klein, S. B. (2012). Self, memory, and the self‐reference effect: An examination of conceptual and methodological issues. Personality and Social Psychology Review: An Official Journal of the Society for Personality and Social Psychology, Inc, 16(3), 283–300. 10.1177/1088868311434214 22291045

[ejn15612-bib-0040] Kok, P. , Jehee, J. F. , & de Lange, F. P. (2012). Less is more: Expectation sharpens representations in the primary visual cortex. Neuron, 75(2), 265–270. 10.1016/j.neuron.2012.04.034 22841311

[ejn15612-bib-0041] Kragel, P. A. , Reddan, M. C. , LaBar, K. S. , & Wager, T. D. (2019). Emotion schemas are embedded in the human visual system. Science Advances, 5(7), eaaw4358. 10.1126/sciadv.aaw4358 31355334 PMC6656543

[ejn15612-bib-0042] Lee, N. A. , Martin, D. , & Sui, J. (2021). A pre‐existing self‐referential anchor is not necessary for self‐prioritisation. Acta Psychologica, 219, 103362. 10.1016/j.actpsy.2021.103362 34273602

[ejn15612-bib-0043] Liang, Q. , Zhang, B. , Fu, S. , Sui, J. , & Wang, F. (2021). The roles of the LpSTS and DLPFC in self‐prioritization: A transcranial magnetic stimulation study. Human Brain MappingAdvance online publication. 10.1002/hbm.25730 PMC8837583 34826160

[ejn15612-bib-0044] Lindquist, K. A. , Wager, T. D. , Kober, H. , Bliss‐Moreau, E. , & Barrett, L. F. (2012). The brain basis of emotion: A meta‐analytic review. The Behavioral and Brain Sciences, 35(3), 121–143. 10.1017/S0140525X11000446 22617651 PMC4329228

[ejn15612-bib-0045] Macrae, C. N. , Visokomogilski, A. , Golubickis, M. , Cunningham, W. A. , & Sahraie, A. (2017). Self‐relevance prioritizes access to visual awareness. Journal of Experimental Psychology. Human Perception and Performance, 43(3), 438–443. 10.1037/xhp0000361 28240929

[ejn15612-bib-0046] McIvor, C. , Sui, J. , Malhotra, T. , Drury, D. , & Kumar, S. (2021). Self‐referential processing and emotion context insensitivity in major depressive disorder. European Journal of Neuroscience, 53(1), 311–329. 10.1111/ejn.14782 32416036

[ejn15612-bib-0047] Menon, V. , & Uddin, L. Q. (2010). Saliency, switching, attention and control: A network model of insula function. Brain Structure & Function, 214(5–6), 655–667. 10.1007/s00429-010-0262-0 20512370 PMC2899886

[ejn15612-bib-0048] Meyer, M. L. , & Lieberman, M. D. (2018). Why people are always thinking about themselves: Medial prefrontal cortex activity during rest primes self‐referential processing. Journal of Cognitive Neuroscience, 30(5), 714–721. 10.1162/jocn_a_01232 29308983

[ejn15612-bib-0049] Modinos, G. , Ormel, J. , & Aleman, A. (2009). Activation of anterior insula during self‐reflection. PLoS ONE, 4(2), e4618. 10.1371/journal.pone.0004618 19242539 PMC2643476

[ejn15612-bib-0050] Molnar‐Szakacs, I. , & Uddin, L. Q. (2013). Self‐processing and the default mode network: Interactions with the mirror neuron system. Frontiers in Human Neuroscience, 7, 571. 10.3389/fnhum.2013.00571 24062671 PMC3769892

[ejn15612-bib-0051] Murphy, K. , Birn, R. M. , Handwerker, D. A. , Jones, T. B. , & Bandettini, P. A. (2009). The impact of global signal regression on resting state correlations: Are anti‐correlated networks introduced? NeuroImage, 44(3), 893–905. 10.1016/j.neuroimage.2008.09.036 18976716 PMC2750906

[ejn15612-bib-0052] Northoff, G. (2016). How does the 'rest‐self overlap' mediate the qualitative and automatic features of self‐reference? Cognitive Neuroscience, 7(1–4), 18–20. 10.1080/17588928.2015.1075483 26317367

[ejn15612-bib-0053] Northoff, G. (2016b). Is the self a higher‐order or fundamental function of the brain? The "basis model of self‐specificity" and its encoding by the brain's spontaneous activity. Cognitive Neuroscience, 7(1–4), 203–222. 10.1080/17588928.2015.1111868 26505808

[ejn15612-bib-0054] Northoff, G. , & Bermpohl, F. (2004). Cortical midline structures and the self. Trends in Cognitive Sciences, 8(3), 102–107. 10.1016/j.tics.2004.01.004 15301749

[ejn15612-bib-0055] Northoff, G. , Schneider, F. , Rotte, M. , Matthiae, C. , Tempelmann, C. , Wiebking, C. , Bermpohl, F. , Heinzel, A. , Danos, P. , Heinze, H. J. , Bogerts, B. , Walter, M. , & Panksepp, J. (2009). Differential parametric modulation of self‐relatedness and emotions in different brain regions. Human Brain Mapping, 30(2), 369–382. 10.1002/hbm.20510 18064583 PMC6870760

[ejn15612-bib-0056] Oosterwijk, S. , Snoek, L. , Rotteveel, M. , Feldman Barrett, L. , & Scholte, S. (2017). Shared states: Using MVPA to test neural overlap between self‐focused emotion imagery and other‐focused emotion understanding. Social Cognitive and Affective Neuroscience, 12(7), 1025–1035. 10.1093/scan/nsx037 28475756 PMC5490677

[ejn15612-bib-0057] Oyserman, D. , Elmore, K. , & Smith, G. (2012). Self, self‐concept, and identity. In M. R. Leary & J. P. Tangney (Eds.), Handbook of self and identity (pp. 69–104). The Guilford Press.

[ejn15612-bib-0058] Perini, I. , Gustafsson, P. A. , Hamilton, J. P. , Kämpe, R. , Zetterqvist, M. , & Heilig, M. (2018). The salience of self, not social pain, is encoded by dorsal anterior cingulate and insula. Scientific Reports, 8, 6165. 10.1038/s41598-018-24658-8 29670166 PMC5906579

[ejn15612-bib-0059] Petersen, S. E. , & Sporns, O. (2015). Brain networks and cognitive architectures. Neuron, 88(1), 207–219. 10.1016/j.neuron.2015.09.027 26447582 PMC4598639

[ejn15612-bib-0060] Power, J. D. , Cohen, A. L. , Nelson, S. M. , Wig, G. S. , Barnes, K. A. , Church, J. A. , Vogel, A. C. , Laumann, T. O. , Miezin, F. M. , Schlaggar, B. L. , & Petersen, S. E. (2011). Functional network organization of the human brain. Neuron, 72(4), 665–678. 10.1016/j.neuron.2011.09.006 22099467 PMC3222858

[ejn15612-bib-0061] Power, J. D. , Mitra, A. , Laumann, T. O. , Snyder, A. Z. , Schlaggar, B. L. , & Petersen, S. E. (2014). Methods to detect, characterize, and remove motion artifact in resting state fMRI. NeuroImage, 84, 320–341. 10.1016/j.neuroimage.2013.08.048 23994314 PMC3849338

[ejn15612-bib-0062] Qiao‐Tasserit, E. , Garcia Quesada, M. , Antico, L. , Bavelier, D. , Vuilleumier, P. , & Pichon, S. (2017). Transient emotional events and individual affective traits affect emotion recognition in a perceptual decision‐making task. PLoS ONE, 12(2), e0171375. 10.1371/journal.pone.0171375 28151976 PMC5289590

[ejn15612-bib-0063] Qin, P. , & Northoff, G. (2011). How is our self related to midline regions and the default‐mode network? NeuroImage, 57(3), 1221–1233. 10.1016/j.neuroimage.2011.05.028 21609772

[ejn15612-bib-0064] Qin, P. , Wang, M. , & Northoff, G. (2020). Linking bodily, environmental and mental states in the self‐a three‐level model based on a meta‐analysis. Neuroscience and Biobehavioral Reviews, 115, 77–95. 10.1016/j.neubiorev.2020.05.004 32492474

[ejn15612-bib-0065] Sander, D. , Grandjean, D. , & Scherer, K. R. (2018). An appraisal‐driven componential approach to the emotional brain. Emotion Review, 10(3), 219–231. 10.1177/1754073918765653

[ejn15612-bib-0066] Scalabrini, A. , Vai, B. , Poletti, S. , Damiani, S. , Mucci, C. , Colombo, C. , Zanardi, R. , Benedetti, F. , & Northoff, G. (2020). All roads lead to the default‐mode network‐global source of DMN abnormalities in major depressive disorder. Neuropsychopharmacology: Official Publication of the American College of Neuropsychopharmacology, 45(12), 2058–2069. 10.1038/s41386-020-0785-x 32740651 PMC7547732

[ejn15612-bib-0067] Schäfer, S. , Wentura, D. , & Frings, C. (2015). Self‐prioritization beyond perception. Experimental Psychology, 62(6), 415–425. 10.1027/1618-3169/a000307 27120563

[ejn15612-bib-0068] Shadlen, M. N. , Britten, K. H. , Newsome, W. T. , & Movshon, J. A. (1996). A computational analysis of the relationship between neuronal and behavioral responses to visual motion. The Journal of Neuroscience, 16(4), 1486–1510. 10.1523/JNEUROSCI.16-04-01486.1996 8778300 PMC6578557

[ejn15612-bib-0069] Sheline, Y. I. , Price, J. L. , Yan, Z. , & Mintun, M. A. (2010). Resting‐state functional MRI in depression unmasks increased connectivity between networks via the dorsal nexus. Proceedings of the National Academy of Sciences of the United States of America, 107(24), 11020–11025. 10.1073/pnas.1000446107 20534464 PMC2890754

[ejn15612-bib-0070] Shi, G. , Li, X. , Zhu, Y. , Shang, R. , Sun, Y. , Wang, H. , Guo, H. , & Sui, J. (2021). The divided brain: Functional brain asymmetry underlying self‐construal. NeuroImage, 240, 118382. 10.1016/j.neuroimage.2021.118382 34252524

[ejn15612-bib-0071] Smith, R. , Lane, R. D. , Alkozei, A. , Bao, J. , Smith, C. , Sanova, A. , Nettles, M. , & Killgore, W. (2018). The role of medial prefrontal cortex in the working memory maintenance of one's own emotional responses. Scientific Reports, 8(1), 3460. 10.1038/s41598-018-21896-8 29472625 PMC5823866

[ejn15612-bib-0072] Smith, S. M. , Fox, P. T. , Miller, K. L. , Glahn, D. C. , Fox, P. M. , Mackay, C. E. , Filippini, N. , Watkins, K. E. , Toro, R. , Laird, A. R. , & Beckmann, C. F. (2009). Correspondence of the brain's functional architecture during activation and rest. Proceedings of the National Academy of Sciences of the United States of America, 106(31), 13040–13045. 10.1073/pnas.0905267106 19620724 PMC2722273

[ejn15612-bib-0073] Soares, A. P. , Macedo, J. , Oliveira, H. M. , Lages, A. , Hernández‐Cabrera, J. , & Pinheiro, A. P. (2019). Self‐reference is a fast‐acting automatic mechanism on emotional word processing: Evidence from a masked priming affective categorisation task. Journal of Cognitive Psychology, 31(3), 317–325. 10.1080/20445911.2019.1599003

[ejn15612-bib-0074] Sridharan, D. , Levitin, D. J. , & Menon, V. (2008). A critical role for the right fronto‐insular cortex in switching between central‐executive and default‐mode networks. Proceedings of the National Academy of Sciences of the United States of America, 105(34), 12569–12574. 10.1073/pnas.0800005105 18723676 PMC2527952

[ejn15612-bib-0075] Sui, J. (2016). Self‐reference acts as a golden thread in binding. Trends in Cognitive Sciences, 20, 482–483. 10.1016/j.tics.2016.04.005 27315761 PMC6029663

[ejn15612-bib-0076] Sui, J. , & Gu, X. (2017). Self as object: Emerging trends in self research. Trends in Neurosciences, 40(11), 643–653. 10.1016/j.tins.2017.09.002 28988827

[ejn15612-bib-0077] Sui, J. , & Humphreys, G. W. (2017). The ubiquitous self: What the properties of self‐bias tell us about the self. Annals of the New York Academy of Sciences, 1396(1), 222–235. 10.1111/nyas.13197 27918835 PMC6029667

[ejn15612-bib-0078] Sui, J. , & Rotshtein, P. (2019). Self‐prioritization and the attentional systems. Current Opinion in Psychology, 29, 148–152. 10.1016/j.copsyc.2019.02.010 30913475

[ejn15612-bib-0079] Sui, J. , Rotshtein, P. , & Humphreys, G. W. (2013). Coupling social attention to the self forms a network for personal significance. Proceedings of the National Academy of Sciences of the United States of America, 110(19), 7607–7612. 10.1073/pnas.1221862110 23610386 PMC3651422

[ejn15612-bib-0080] Sun, Y. , Fuentes, L. J. , Humphreys, G. W. , & Sui, J. (2016). Dataset of embodied perspective enhances self and friend‐biases in perceptual matching. Data in Brief, 8, 1374–1376. 10.1016/j.dib.2016.06.062 27583343 PMC4993850

[ejn15612-bib-0081] Thomason, M. E. , Chang, C. E. , Glover, G. H. , Gabrieli, J. D. , Greicius, M. D. , & Gotlib, I. H. (2008). Default‐mode function and task‐induced deactivation have overlapping brain substrates in children. NeuroImage, 41(4), 1493–1503. 10.1016/j.neuroimage.2008.03.029 18482851 PMC2735193

[ejn15612-bib-0082] Tomasi, D. , & Volkow, N. D. (2011). Functional connectivity hubs in the human brain. NeuroImage, 57(3), 908–917. 10.1016/j.neuroimage.2011.05.024 21609769 PMC3129362

[ejn15612-bib-0083] Touroutoglou, A. , Lindquist, K. A. , Dickerson, B. C. , & Barrett, L. F. (2015). Intrinsic connectivity in the human brain does not reveal networks for 'basic' emotions. Social Cognitive and Affective Neuroscience, 10(9), 1257–1265. 10.1093/scan/nsv013 25680990 PMC4560947

[ejn15612-bib-0084] Uddin, L. Q. (2015). Salience processing and insular cortical function and dysfunction. Nature Reviews. Neuroscience, 16(1), 55–61. 10.1038/nrn3857 25406711

[ejn15612-bib-0085] Uddin, L. Q. , Iacoboni, M. , Lange, C. , & Keenan, J. P. (2007). The self and social cognition: The role of cortical midline structures and mirror neurons. Trends in Cognitive Sciences, 11(4), 153–157. 10.1016/j.tics.2007.01.001 17300981

[ejn15612-bib-0086] Uddin, L. Q. , Nomi, J. S. , Hébert‐Seropian, B. , Ghaziri, J. , & Boucher, O. (2017). Structure and function of the human insula. Journal of Clinical Neurophysiology: Official Publication of the American Electroencephalographic Society, 34(4), 300–306. 10.1097/WNP.0000000000000377 28644199 PMC6032992

[ejn15612-bib-0087] Uddin, L. Q. , Yeo, B. , & Spreng, R. N. (2019). Towards a universal taxonomy of macro‐scale functional human brain networks. Brain Topography, 32(6), 926–942. 10.1007/s10548-019-00744-6 31707621 PMC7325607

[ejn15612-bib-0088] Ursino, M. , Ricci, G. , & Magosso, E. (2020). Transfer entropy as a measure of brain connectivity: A critical analysis with the help of neural mass models. Frontiers in Computational Neuroscience, 14, 45. 10.3389/fncom.2020.00045 32581756 PMC7292208

[ejn15612-bib-0089] van Buuren, M. , Walsh, R. J. , Sijtsma, H. , Hollarek, M. , Lee, N. C. , Bos, P. A. , & Krabbendam, L. (2020). Neural correlates of self‐ and other‐referential processing in young adolescents and the effects of testosterone and peer similarity. NeuroImage, 219, 117060. 10.1016/j.neuroimage.2020.117060 32561475

[ejn15612-bib-0090] van den Heuvel, M. P. , Mandl, R. C. , Kahn, R. S. , & Hulshoff Pol, H. E. (2009). Functionally linked resting‐state networks reflect the underlying structural connectivity architecture of the human brain. Human Brain Mapping, 30(10), 3127–3141. 10.1002/hbm.20737 19235882 PMC6870902

[ejn15612-bib-0091] van der Meer, L. , Costafreda, S. , Aleman, A. , & David, A. S. (2010). Self‐reflection and the brain: A theoretical review and meta‐analysis of neuroimaging studies with implications for schizophrenia. Neuroscience and Biobehavioral Reviews, 34(6), 935–946. 10.1016/j.neubiorev.2009.12.004 20015455

[ejn15612-bib-0092] Van Dijk, K. R. , Sabuncu, M. R. , & Buckner, R. L. (2012). The influence of head motion on intrinsic functional connectivity MRI. NeuroImage, 59(1), 431–438. 10.1016/j.neuroimage.2011.07.044 21810475 PMC3683830

[ejn15612-bib-0093] Vatansever, D. , Menon, D. K. , & Stamatakis, E. A. (2017). Default mode contributions to automated information processing. Proceedings of the National Academy of Sciences of the United States of America, 114(48), 12821–12826. 10.1073/pnas.1710521114 29078345 PMC5715758

[ejn15612-bib-0094] Verplanken, B. , & Sui, J. (2019). Habit and identity: Behavioural, cognitive, affective, and motivational facets of an integrated self. Frontiers in Psychology, 10, 1504. 10.3389/fpsyg.2019.01504 31354563 PMC6635880

[ejn15612-bib-0095] Wagner, D. D. , Haxby, J. V. , & Heatherton, T. F. (2012). The representation of self and person knowledge in the medial prefrontal cortex. Wiley Interdisciplinary Reviews: Cognitive Science, 3(4), 451–470. 10.1002/wcs.1183 22712038 PMC3375705

[ejn15612-bib-0096] Wang, H. , Humphreys, G. , & Sui, J. (2016). Expanding and retracting from the self: Gains and costs in switching self‐associations. Journal of Experimental Psychology: Human Perception and Performance, 42(2), 247–256. 10.1037/xhp0000125 26348068 PMC4730907

[ejn15612-bib-0097] Whitfield‐Gabrieli, S. , Moran, J. M. , Nieto‐Castañón, A. , Triantafyllou, C. , Saxe, R. , & Gabrieli, J. D. (2011). Associations and dissociations between default and self‐reference networks in the human brain. NeuroImage, 55(1), 225–232. 10.1016/j.neuroimage.2010.11.048 21111832

[ejn15612-bib-0098] Yankouskaya, A. , Bührle, R. , Lugt, E. , Stolte, M. , & Sui, J. (2020). Intertwining personal and reward relevance: Evidence from the drift‐diffusion model. Psychological Research, 84(1), 32–50. 10.1007/s00426-018-0979-6 29368227

[ejn15612-bib-0099] Yankouskaya, A. , Humphreys, G. , Stolte, M. , Stokes, M. , Moradi, Z. , & Sui, J. (2017). An anterior‐posterior axis within the ventromedial prefrontal cortex separates self and reward. Social Cognitive and Affective Neuroscience, 12(12), 1859–1868. 10.1093/scan/nsx112 29040796 PMC5716107

[ejn15612-bib-0100] Yankouskaya, A. , Palmer, D. , Stolte, M. , Sui, J. , & Humphreys, G. W. (2017). Self‐bias modulates saccadic control. Quarterly Journal of Experimental Psychology (2006), 70(12), 2577–2585. 10.1080/17470218.2016.1247897 27739335

[ejn15612-bib-0101] Yankouskaya, A. , & Sui, J. (2021). Self‐positivity or self‐negativity as a function of the medial prefrontal cortex. Brain Sciences, 11(2), 264. 10.3390/brainsci11020264 33669682 PMC7922957

[ejn15612-bib-0102] Yaoi, K. , Osaka, M. , & Osaka, N. (2015). Neural correlates of the self‐reference effect: Evidence from evaluation and recognition processes. Frontiers in Human Neuroscience, 9, 383. 10.3389/fnhum.2015.00383 26167149 PMC4481146

[ejn15612-bib-0103] Yin, S. , Bi, T. , Chen, A. , & Egner, T. (2021). Ventromedial prefrontal cortex drives the prioritization of self‐associated stimuli in working memory. Journal of Neuroscience, 41(9), 2012–2023. 10.1523/JNEUROSCI.1783-20.2020 33462089 PMC7939096

[ejn15612-bib-0104] Zalesky, A. , Fornito, A. , & Bullmore, E. T. (2010). Network‐based statistic: Identifying differences in brain networks. NeuroImage, 53(4), 1197–1207. 10.1016/j.neuroimage.2010.06.041 20600983

[ejn15612-bib-0105] Zhang, W. , Li, H. , & Pan, X. (2015). Positive and negative affective processing exhibit dissociable functional hubs during the viewing of affective pictures. Human Brain Mapping, 36(2), 415–426. 10.1002/hbm.22636 25220389 PMC6869282

[ejn15612-bib-0106] Zhou, Z. , Chen, Y. , Ding, M. , Wright, P. , Lu, Z. , & Liu, Y. (2009). Analyzing brain networks with PCA and conditional granger causality. Human Brain Mapping, 30(7), 2197–2206. 10.1002/hbm.20661 18830956 PMC6871256

[ejn15612-bib-0107] Zhu, Y. , Li, X. , Sun, Y. , Wang, H. , Guo, H. , & Sui, J. (2021). Investigating neural substrates of individual Independence and interdependence orientations via efficiency‐based dynamic functional connectivity: A machine learning approach. IEEE Transaction on Cognitive Developmental Systems. 10.1109/TCDS.2021.3101643

