# Supplementary material for: Self‐prioritization is supported by interactions between large‐scale brain networks
Source: Eur J Neurosci. 2022 Feb 3;55(5):1244–61. doi: 10.1111/ejn.15612 (PMC9303922; doi:10.1111/ejn.15612)
Supplement: Supplementary file 1 — Table S1. Summary of datasets and behavioural results. Table S2. Comparison key findings* with** and without Global Signal regression (GSR). All contrasts were defined using ‘height’ threshold of p < 0.001 (uncorrected) and cluster‐corrected p‐FWE < 0.05 Table S3. The results of Network Based Statistic analysis with varied ‘height’ thresholds for contrast [ self > stranger ] (p‐value for each component was FWE corrected). NS denotes that a component did not survive the FWE correction. Table S4. The results of Network Based Statistic analysis with varied ‘height’ thresholds for contrast [ self > friend ] (p‐value for each component was FWE corrected). NS denotes that a component did not survive the FWE correction. Table S5. The results of Network Based Statistic analysis with varied ‘height’ thresholds for contrast [ sad > neutral ] (p‐value for each component was FWE corrected). NS denotes that a component did not survive the FWE correction. Table S6. The results of Network Based Statistic analysis with varied ‘height’ thresholds for contrast [ happy > neutral ] (p‐value for each component was FWE corrected). NS denotes that a component did not survive the FWE correction. Table S7. The results of Network Based Statistic analysis with varied ‘height’ thresholds for contrast [ self‐bias > sad‐bias ] (p‐value for each component was FWE corrected). NS denotes that a component did not survive the FWE correction. Table S8. The results of Network Based Statistic analysis with varied ‘height’ thresholds for contrast [ self‐bias > happy‐bias ] (p‐value for each component was FWE corrected). NS denotes that a component did not survive the FWE correction. Table S9. The results of Network Based Statistic analysis with varied ‘height’ thresholds for contrast [ self > friend ] in validation data set (p‐value for each component was FWE corrected). NS denotes that a component did not survive the FWE correction. [file EJN-55-1244-s001.docx]

**Supplementary Materials**

**Self-prioritization is supported by interactions between large-scale brain networks**

Table S1. Summary of datasets and behavioural results.

| **Study 1 (Yankouskaya & Sui, 2021*)** | **Study 2 (Yankouskaya et al., 2017**)** |
| --- | --- |
| ***Participants***  In both studies, participants reported no use of psychotropic medications or past diagnoses for psychiatric, neurological disorders and have normal or corrected-to-normal vision size. | |
| Twenty-one young, healthy adults aged between 21-26 (10 males, age M=23.6, SD =2.8). As a part of the pre-screening procedure, participants performed Mood and Anxiety Symptom Questionnaire (MASQ), a 77-items self-report questionnaire that assesses depressive, anxious and mixed symptomatology. Only participants with low scores on each of 5 subscales were invited to the scanning session. | Sixteen healthy volunteers aged between 22-34 (8 males, age M = 26.1, SD = 7.5). |
| ***Stimuli***  Six geometric shapes (circle, hexagon, square, rectangle, diamond and triangle) were randomly assigned to three conditions in each task. A stimulus display contained a fixation cross (0.7°×0.7°) on the center of the screen with a shape (covering 3.5° × 3.5° of visual angle) and label (or a schematic face) covering 1.76°/ 2.52° × 1.76° (3.5° × 3.5°) of visual angle on either side of fixation. The distance between the shape and the label (or a schematic face) was 10 degrees. Left-right presentations of the shapes and labels/schematic faces were counterbalanced across trials. Each trial started with a fixation cross for 200 ms, followed by the stimulus display for 100 ms and a blank interval which remained for 1000 ms or until the participant responded. Trials were separated by a jittered interstimulus interval (ranging between 2500-6000 ms). | |
| ***Trial number*** | |
| Five runs of 72 trials in each task | Four runs of 48 trials |
| ***Imaging data acquisition*** | |
| Structural and functional images were acquired at Nuffield Department of Clinical Neurosciences (FMRIB, Oxford, UK) on a 3-Tesla whole-body scanner (Siemens Magnetom Prisma) and a standard 32 channel coil. Tasks functional volumes were acquired using an interleaved, gradient-echo echo-planar pulse sequence with the following parameters with a gradient echo T2*-weighted echo-planar sequence (TR 2040 ms, TE 30 ms, flip angle 80, 64 × 64 matrix, field of view 192 mm, voxel size 3x3x3mm, parallel imaging GRAPPA, bandwidth = 1628 Hz/Px, PE = 2, and interleaved slice ordering). A total of 36 axial slices (3 mm thick, no gap) were sampled for whole-brain coverage excluding the cerebellum. Data were acquired in five runs of 180 volumes each. Each run lasted approximately 5 min 10 sec. Whole-brain anatomical images were acquired using a T1-weighted high-resolution magnetization prepared gradient echo (MPRAGE) sequence: TR = 1900 ms, TE = 3.97 ms, flip angle = 8°; field of view (FOV) = 192 mm, voxel size 1 × 1 × 1 mm. | Structural and functional images were acquired at the Nuffield Department of Clinical Neurosciences (FMRIB, Oxford, UK) on a 3T scanner (Trio, Siemens) using a 24-channel head coil. Task functional images were acquired with a gradient echo T2*-weighted echo-planar sequence (TR 2000 ms, TE 30 ms, flip angle 70, 64x64 matrix, field of view 19.2^2^ mm, voxel size 3x3x3mm). A total of 36 axial slices (3 mm thick, no gap) were sampled for whole-brain coverage excluding the cerebellum. Imaging data were acquired in four separate 120-volume runs of 4 min 02 s each. A high-resolution T1-weighted anatomical scan of the whole brain was acquired (256 x 256 matrix, voxel size 1 x 1 x 1 mm, TR = 1900 ms, TE = 3.97 ms, flip angle = 8°). |
| ***Main behavioural results***  In both studies, participants were accurate in responding to stimuli (percent of correct responses varied from 82 to 94). The task associative task generated robust self-prioritization effects. | |
| In the personal task, participants were faster (F(2,40)= 27.62, p<0.001) in responding to stimuli associated with self and friend compared to stranger (*t*(20)= -7.32, p<0.001, MD= -88.24, 95% CI [-100.1; -72.71]; *t*(20)= -4.78, p < 0.001, MD= -57.66, 95% CI [-82.98; -32.33] respectively). The difference between self and friend was also significant (*t*(20) = -2.54, p=0.02, MD = -30.58, 95%CI [-60.37,-2.84].  In the emotion task, reaction times for happy and sad associations were faster compared to associations with neutral emotional expression (F(2,40)=29.70, p<0.001; *t*(20)= -6.83, p<0.001, MD = -69.38, 95%CI [-84.93, -47.35]); *t*(20)= -6.51, p<0.001, MD= -66.14, 95% CI [-82.97; -47.78]). The difference between happy and sad associations were not significant (*t*(20)= -0.32, p=0.75). | In the personal task, participants were faster for stimuli associated with self compared to stimuli associated with friend (t(15) =-4.44, p<.001. MD = -101.99, 95% CI [=151.31, -52.62], Cohen’s d = -1.15. |

* Yankouskaya, A., & Sui, J. (2021). Self-Positivity or Self-Negativity as a Function of the Medial Prefrontal Cortex. *Brain sciences*, *11*(2), 264. https://doi.org/10.3390/brainsci11020264

**Yankouskaya, A., Humphreys, G., Stolte, M., Stokes, M., Moradi, Z., & Sui, J. (2017). An anterior-posterior axis within the ventromedial prefrontal cortex separates self and reward. *Social cognitive and affective neuroscience*, *12*(12), 1859–1868. <https://doi.org/10.1093/scan/nsx112>

Table S2. Comparison key findings* with** and without Global Signal regression (GSR). All contrasts were defined using ‘height’ threshold of p < .001 (uncorrected) and cluster-corrected p-FWE < .05

| NBS without GSR | NBS with GSR |
| --- | --- |
| Self-prioritization effect (contrast [self > stranger]: mass 90.64, p-FWE = .009, size 4 (DMN.MPFC, SN.AI_left, SN.AI_right, FPN.LPFC | Self-prioritization effect (contrast [self > stranger]: mass 84.65, p-FWE = .01, size 4  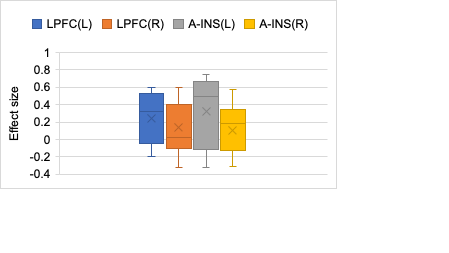  The cluster retained the same connections. However, the effects sizes for individual connections was reduces (especially, for connectivity between DMN.MPFC and SN.AI_right |
| Self-prioritization effect, contrast [self > friend]: mass = 48.99, p-FDR = .048, p-FWE = .058; size = 2 | mass = 23.12, p-FDR = .07, p-FWE = .09; size = 2 |
| Negative emotion bias (contrast [sad > neutral]): mass = 82.91, p-FWE = .013; size = 4 (DMN.MPFC, VisMedial, DAN.FEF_left, DAN.FEF_right | mass = 76.22, p-FWE = .024; size = 3  Compared to the results without GSR, the cluster was reduced in size. Reducing in size was due to instead of bidirectional connections between DMN.MPFC and VisMedia, here we observed only connection from VisMed to DMN.MPFC. The effect size for this connection was reduced to negligible (EZ=0.03) |
| Positive emotion-prioritization, contrast [happy > neutral]: mass = 56.50, p-FWE = .034; size = 2 (DMN.MPFC, SN.RPFC_left, FPN.PPC_left) | mass = 52.50, p-FWE = .046; size = 2  (DMN.MPFC, SN.RPFC_left, FPN.PPC_left). There was no reduction in size of the cluster. However the effect sizes decreased for all individual connections |
| Contrast [self-prioritization > sad-prioritization]: mass = 198.91, p-FEW <.001, size = 8 (DMN.MPFC, FPN.PPC_left, FPN.PPC_right, DAN_FEF_left, DAN.FEF_right, SN.AI_left, SN.AI_right, DMN.LP_left | Contrast [self-prioritization > sad-prioritization]: mass = 147.81, p-FEW <.001, size = 7. There was reduction in mass associated with reduction in individual connections effect sizes. Also, one connection (between DMN.MPFC and DMN.LP_left) was not longer identified withing the cluster  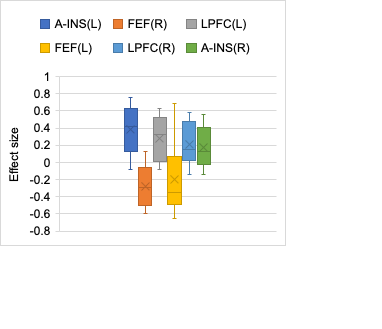 |
| Contrast [self-prioritization > happy-prioritization]: mass = 99.21, size = 4, p-FWE = .007 (DMN.MPFC, FPN.LPFC_left, FPN.PPC_left, SN.RPFC_left, SN.AI_left) | Contrast [self-prioritization > happy-prioritization]: mass = 64.03, size = 4, p-FWE = .01 (DMN.MPFC, FPN.LPFC_left, FPN.PPC_left, SN.RPFC_left, SN.AI_left).  There was no reduction in the cluster size. However, there was a reduction in effect sizes of individual connections  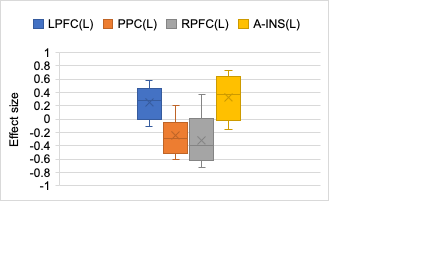 |
| Discussion | |
| Overall, the functional connectivity results with GSR were close to our original results. Although we did not tested the effect of pre-processing type on functional connectivity, the result in Table 2 indicate general decrease in connectivity when GSR was applied. The reduction in effect sizes in the present study indicates GSR affected within-node connectivity. This finding is in line with multiple studies showing that the connectivity decreases after GSR (Chang & Glover, 2009; Fox et al., 2009; Weissenbacjer et al., 2009). | |

*The results of Network Based Statistics (NBS) analysis

**To estimate functional connectivity with GSR, we added a new ROI that encompass the entire brain and entered this ROI into the confounds list in the preprocessing step. The CompCor procedure implemented in CONN as an alternative to GSR was not used in this case.

Table S3. The results of Network Based Statistic analysis with varied ‘height’ thresholds for contrast [***self > stranger***] (p-value for each component was FWE corrected). NS denotes that a component did not survive the FWE correction.

| Height threshold  (p< ) | Connections comprising a component | Component statistics | | |
| --- | --- | --- | --- | --- |
|  |  | mass | size | p-FWE  value |
| .05 - .01 | NS |  |  |  |
| .009 - .004 | NS |  |  |  |
| .003 - .001 | DMN.MPFC->Salience.A-INS(L)  DMN.MPFC->Salience.A-INS(R)  DMN.MPFC->Frontoparietal.LPFC(L)  DMN.MPFC->Frontoparietal.LPFC(R) | 90.64 | 4 | .029 - .009 |
| .0009 - .0007 | DMN.MPFC->Salience.A-INS(L)  DMN.MPFC->Salience.A-INS(R)  DMN.MPFC->Frontoparietal.LPFC(L)  DMN.MPFC->Frontoparietal.LPFC(R) | 90.64 | 4 | .006 -.004 |
| .0006 - .0001 | DMN.MPFC->Salience.A-INS(L)  DMN.MPFC->Frontoparietal.LPFC(L) | 58.04 | 2 | .025 - .013 |
| .00009 - .00006 | DMN.MPFC->Salience.A-INS(L)  DMN.MPFC->Frontoparietal.LPFC(L) | 58.04 | 2 | .013 -.014 |
| .00005 - .00002 | DMN.MPFC->Frontoparietal.LPFC(L) | 32.10 | 1 | .021 - .024 |
| .00001 | NS |  |  |  |

Table S4. The results of Network Based Statistic analysis with varied ‘height’ thresholds for contrast [***self > friend***] (p-value for each component was FWE corrected). NS denotes that a component did not survive the FWE correction.

| Height threshold  (p< ) | Connections comprising a component | Component statistics | | |
| --- | --- | --- | --- | --- |
|  |  | mass | size | p-FWE  value |
| .05 - .01 | NS |  |  |  |
| .009 | DMN.MPFC->Salience.A-INS(L)  DMN.MPFC->Frontoparietal.LPFC(L)  DMN.MPFC-> Frontoparietal.LPFC(R)  Salience.STG(R) -> Frontoparietal.PPC(R)  Frontoparietal.PPC(R)-> Salience.STG(R)  Frontoparietal.PPC(R)->Salience.RFPC(R)  Salience.RFPC(R)-> Frontoparietal.PPC(R)  Salience.RFPC(R)-> Frontoparietal.LPFC(R)  Frontoparietal.LPFC(R)-> Salience.RFPC(R)  Salience.A-INS(L)-> Frontoparietal.PPC(R)  Frontoparietal.PPC(R)-> Salience.A-INS(L) | 130.65 | 11 | .049 |
| .008 - .002 | NS |  |  |  |
| .001 - .0001 | DMN.MPFC->Salience.A-INS(L)  DMN.MPFC->Frontoparietal.LPFC(L) | 48.99 | 2 | .050 - .015 |
| .00009 | DMN.MPFC->Salience.A-INS(L)  DMN.MPFC->Frontoparietal.LPFC(L) | 58.04 | 2 | .025 |
| .00008 | DMN.MPFC->Salience.A-INS(L) | 24.69 | 1 | .034 |
| .00007 - .00001 | NS |  |  |  |

Table S5. The results of Network Based Statistic analysis with varied ‘height’ thresholds for contrast [***sad > neutral***] (p-value for each component was FWE corrected). NS denotes that a component did not survive the FWE correction.

| Height threshold  (p< ) | Connections comprising a component | Component statistics | | |
| --- | --- | --- | --- | --- |
|  |  | mass | size | p-FWE  value |
| .05 - .01 | NS |  |  |  |
| .009 - .005 | NS |  |  |  |
| .004 - .0006 | DMN.MPFC->DorsalAttention.FEF(L)  DMN.MPFC->DorsalAttention.FEF(R)  DMN.MPFC->Visual.Media  Visual.Media-> DMN.MPFC | 82.91 | 4 | .006 |
| .0005 - .0001 | DMN.MPFC->DorsalAttention.FEF(L)  DMN.MPFC->DorsalAttention.FEF(R) | 54.13 | 2 | .022 |
| .00009 - .00003 | DMN.MPFC->DorsalAttention.FEF(L)  DMN.MPFC->DorsalAttention.FEF(R) | 54.13 | 2 | .007 |
| .00002 | DMN.MPFC->DorsalAttention.FEF(L) | 48.32 | 1 | .007 |
| .00001 | NS |  |  |  |

Table S6. The results of Network Based Statistic analysis with varied ‘height’ thresholds for contrast [***happy > neutral***] (p-value for each component was FWE corrected). NS denotes that a component did not survive the FWE correction.

| Height threshold  (p< ) | Connections comprising a component | Component statistics | | |
| --- | --- | --- | --- | --- |
|  |  | mass | size | p-FWE  value |
| .05 - .01 | NS |  |  |  |
| .009 - .004 | NS |  |  |  |
| .003 - .002 | DMN.MPFC->Salience.RPFC(L)  DMN.MPFC-> Frontoparietal.PPC(L)  DMN.MPFC->Language.pSTG(R) | 78.93 | 2 | .032 |
| .001 - .0001 | DMN.MPFC->Salience.RPFC(L)  DMN.MPFC-> Frontoparietal.PPC(L) | 56.50 | 2 | .034 |
| .00009 - .00003 | DMN.MPFC->Salience.RPFC(L)  DMN.MPFC-> Frontoparietal.PPC(L) | 58.04 | 2 | .034 |
| .00002 | DMN.MPFC-> Frontoparietal.PPC(L) | 29.90 | 1 | .008 |
| .00001 | NS |  |  |  |

Table S7. The results of Network Based Statistic analysis with varied ‘height’ thresholds for contrast [***self-bias > sad-bias***] (p-value for each component was FWE corrected). NS denotes that a component did not survive the FWE correction.

| Height threshold  (p< ) | Connections comprising a component | Component statistics | | |
| --- | --- | --- | --- | --- |
|  |  | mass | size | p-FWE  value |
| .05 | Widely distributed components (detailed information can be found in p<.05_threshold_self-bas>sad_bias.txt | 615.64 | 66 | .024 |
| .04 | Widely distributed components (detailed information can be found in p<.04_threshold_self-bas>sad_bias.txt | 551.70 | 54 | .026 |
| .03 | Widely distributed components (detailed information can be found in p<.03_threshold_self-bas>sad_bias.txt | 510.17 | 46 | .016 |
| .02 | Widely distributed components (detailed information can be found in p<.02_threshold_self-bas>sad_bias.txt | 462.65 | 38 | .007 |
| .01 | Widely distributed components (detailed information can be found in p<.01_threshold_self-bas>sad_bias.txt | 335.93 | 22 | .005 |
| .009 - .003 | DMN.MPFC-> Frontoparietal.LPFC(L)  Frontoparietal.LPFC(L) -> DMN.MPFC  DMN.LP(L) ->Frontoparietal.LPFC(L)  Frontoparietal.LPFC(L)-> DMN.LP(L)  DMN.LP(L) -> Frontoparietal.PPC(L)  Frontoparietal.PPC(L)-> DMN.LP(L)  DMN.MPFC->Salience.AInsula(L)  DMN.MPFC->Dorsal Attention.FEF(L)  DMN.MPFC->Dorsal Attention.FEF(R)  DMN.MPFC->Salience.AInsula(R)  DMN.MPFC-> Frontoparietal.LPFC(R) | 246.08 | 12 | .013 |
| .002 - .0008 | DMN.LP(L)-> Frontoparietal.LPFC(L)  Frontoparietal.LPFC(L) -> DMN.LP(R)  DMN.LP(L) ->Frontoparietal.LPFC(L)  DMN.MPFC->Salience.AInsula(L)  DMN.MPFC->Dorsal Attention.FEF(L)  DMN.MPFC->Dorsal Attention.FEF(R)  DMN.MPFC-> Frontoparietal.LPFC(R)  DMN.MPFC->Salience.AInsula(R) | 198.91 | 8 | <.001 |
| .0007 - .0006 | DMN.LP(L)-> Frontoparietal.LPFC(L)  Frontoparietal.LPFC(L) -> DMN.LP(R)  DMN.LP(L) ->Frontoparietal.LPFC(L)  DMN.MPFC->Salience.AInsula(L)  DMN.MPFC->Dorsal Attention.FEF(L)  DMN.MPFC->Dorsal Attention.FEF(R)  DMN.MPFC-> Frontoparietal.LPFC(R) | 183.38 | 7 | .0006 |
| .0005 -.00004 | DMN.LP(L)-> Frontoparietal.LPFC(L)  Frontoparietal.LPFC(L) -> DMN.LP(R)  DMN.LP(L) ->Frontoparietal.LPFC(L)  DMN.MPFC->Salience.AInsula(L)  DMN.MPFC->Dorsal Attention.FEF(L)  DMN.MPFC->Dorsal Attention.FEF(R) | 166.48 | 6 | .001 |
| .00005 | DMN.LP(L)-> Frontoparietal.LPFC(L)  Frontoparietal.LPFC(L) -> DMN.LP(R)  DMN.MPFC->Salience.AInsula(L)  DMN.MPFC->Dorsal Attention.FEF(R)  DMN.MPFC-> Frontoparietal.LPFC(R) | 140.96 | 5 | .002 |
| .00001 | NS |  |  |  |

Table S8. The results of Network Based Statistic analysis with varied ‘height’ thresholds for contrast [***self-bias > happy-bias***] (p-value for each component was FWE corrected). NS denotes that a component did not survive the FWE correction.

| Height threshold  (p< ) | Connections comprising a component | Component statistics | | |
| --- | --- | --- | --- | --- |
|  |  | mass | size | p-FWE  value |
| .05 - .005 | NS |  |  |  |
| .004 - .002 |  |  |  |  |
| .003 - .002 | DMN.MPFC-> Frontoparietal.LPFC(L)  DMN.MPFC-> Frontoparietal.PPC(L)  DMN.MPFC->Salience.RPFC(L)  DMN.MPFC->Salience.AInsula(L)  DMN.MPFC-> Frontoparietal.LPFC(R) | 113.36 | 5 | .010 |
| .001 - .0002 | DMN.MPFC-> Frontoparietal.LPFC(L)  DMN.MPFC-> Frontoparietal.PPC(L)  DMN.MPFC->Salience.RPFC(L)  DMN.MPFC->Salience.AInsula(L) | 99.21 | 4 | .007 |
| .00001 | DMN.MPFC-> Frontoparietal.LPFC(L) | 35.08 | 1 | .042 |
| .000009 | NS |  |  |  |

Table S9. The results of Network Based Statistic analysis with varied ‘height’ thresholds for contrast [***self > friend***] in validation data set (p-value for each component was FWE corrected). NS denotes that a component did not survive the FWE correction.

| Height threshold  (p< ) | Connections comprising a component | Component statistics | | |
| --- | --- | --- | --- | --- |
|  |  | mass | size | p-FWE  value |
| .05 - .03 | NS |  |  |  |
| .02 - .01 | DMN.MPFC->Salience.A-INS(L)  DMN.MPFC->Salience.A-INS(R)  DMN.MPFC->Frontoparietal.LPFC(L)  DMN.MPFC->Frontoparietal.LPFC(R) | 220.92 | 4 | .025-.01 |
| .009 - .001 | DMN.MPFC->Salience.A-INS(L)  DMN.MPFC->Salience.A-INS(R)  DMN.MPFC->Frontoparietal.LPFC(L)  DMN.MPFC->Frontoparietal.LPFC(R) | 220.92 | 4 | <.001 |
| .0009 - .0004 | DMN.MPFC->Salience.A-INS(L)  DMN.MPFC->Salience.A-INS(R)  DMN.MPFC->Frontoparietal.LPFC(L)  DMN.MPFC->Frontoparietal.LPFC(R) | 220.92 | 4 | <.001 |
| .0003 - .0001 | DMN.MPFC->Salience.A-INS(L)  DMN.MPFC->Frontoparietal.LPFC(L)  DMN.MPFC->Salience.A-INS(R) | 199.44 | 3 | .0003-.0008 |
| .00009 - .00006 | DMN.MPFC->Salience.A-INS(L)  DMN.MPFC->Salience.A-INS(R) | 169.38 | 2 | .0006- |
| .00005 - .000009 | DMN.MPFC->Salience.A-INS(L) | 48.11 | 1 | .0003 - .02 |
| .000001 | NS |  |  |  |
